# Improvement in creep life of a nickel-based single-crystal superalloy via composition homogeneity on the multiscales by magnetic-field-assisted directional solidification

**DOI:** 10.1038/s41598-018-19800-5

**Published:** 2018-01-23

**Authors:** Weili Ren, Chunlin Niu, Biao Ding, Yunbo Zhong, Jianbo Yu, Zhongming Ren, Wenqing Liu, Liangpu Ren, Peter K. Liaw

**Affiliations:** 1 State Key Laboratory of Advanced Special Steel, College of Materials Science and Engineering, Shanghai University, Shanghai, 200072 PR China; 2 Instrumental Analysis & Research Center, Shanghai University, Shanghai, 200444 PR China; 3 Microelectric Research and Development Center, Shanghai University, Shanghai, 200072 PR China; 4 Department of Materials Science and Engineering, The University of Tennessee, Knoxville, TN 37996 USA

## Abstract

The improvement of the creep properties of single-crystal superalloys is always strongly motivated by the vast growing demand from the aviation, aerospace, and gas engine. In this study, a static magnetic-field-assisted solidification process significantly improves the creep life of single-crystal superalloys. The mechanism originates from an increase in the composition homogeneity on the multiscales, which further decreases the lattice misfit of γ/γ′ phases and affects the phase precipitation. The phase-precipitation change is reflected as the decrease in the γ′ size and the contents of carbides and γ/γ′ eutectic, which can be further verified by the variation of the cracks number and raft thickness near the fracture surface. The variation of element partition decreases the dislocation quantity within the γ/γ′ phases of the samples during the crept deformation. Though the magnetic field in the study destroys the single-crystal integrity, it does not offset the benefits from the compositional homogeneity. The proposed means shows a great potential application in industry owing to its easy implement. The uncovered mechanism provides a guideline for controlling microstructures and mechanical properties of alloys with multiple components and multiple phases using a magnetic field.

## Introduction

**Figure 1:**
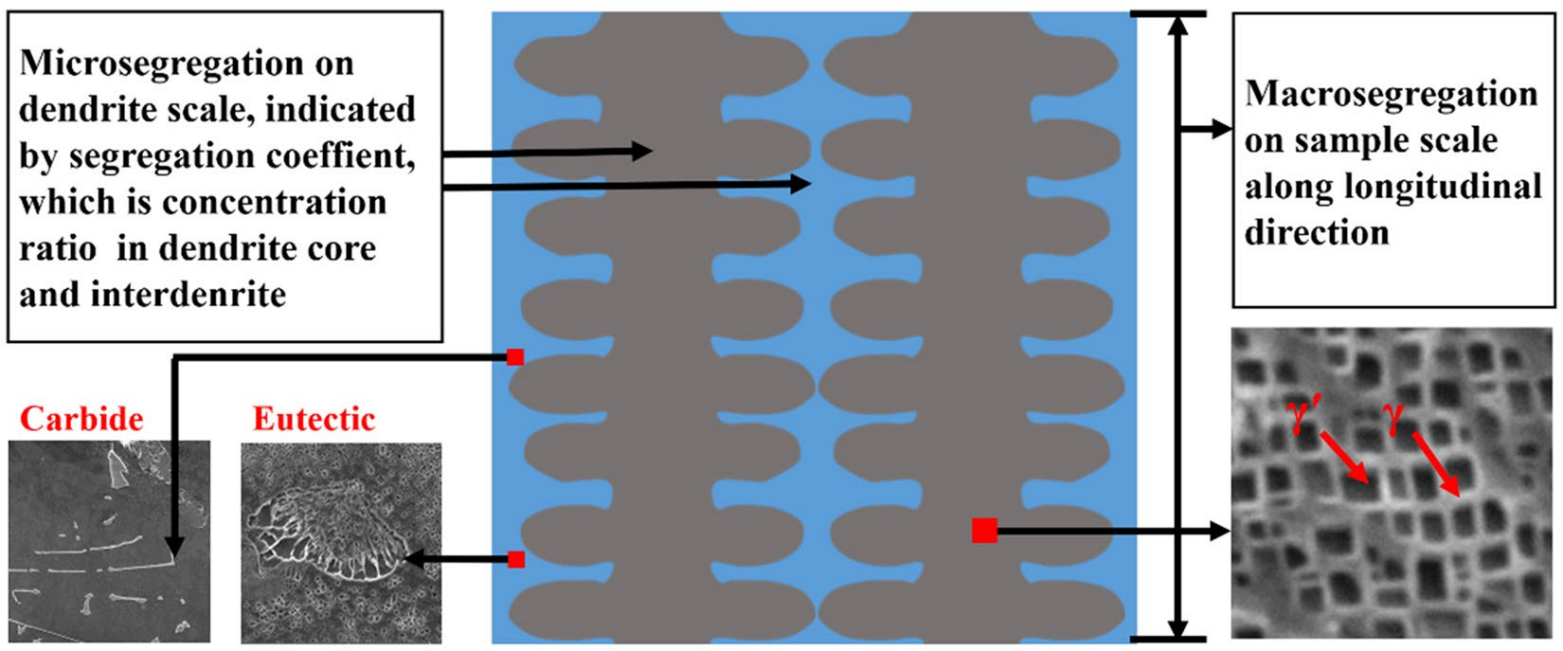
The structure illustration of the single-crystal nickel-based superalloy.

Nickel-based superalloys, especially single-crystal (SC) ones, have long been recognized as important materials for turbine blades used in the aviation, aerospace, and gas engines. The SC superalloys are employed in the advanced turbine blades where high-temperature strength and creep life are required. A vast growing demand for the engine capabilities impels the continuous development of high creep-resistant superalloys^1–3^. The SC superalloys possess a structure of the directional-array dendrites (Fig. 1), where the most strengthening phase, γ′ (Ni_3_Al), is homogeneously distributed in the γ (Ni) matrix. The carbides and γ/γ′ eutectic phases generally locate around the interdendrite area (Fig. 1). The high creep resistance and life is controlled by the SC integrity^4,5^, the arm spacing of the dendrite^6,7^, the content and size of precipitation phase^2,3,8–10^, and the segregation of the alloying elements^10,11^. In order to improve the creep properties of the SC superalloys, the researchers continuously add elements^3,12^ or perfect the solidification processes^7,13–17^ to modify the structures, phases, and defects

.

Through the elemental addition, the SC superalloys have been developed up to the sixth generation^1,9,12^. With the generation development, the contents of the alloying elements gradually increase in order to produce the solid-solution and precipitation-phase strengthening and further achieve the higher strength and creep life. First generation SC superalloys contain no Re and Ru. Second and third generation superalloys contain about 3 and 5 weigh percent (wt.%) Re, respectively. Fourth and fifth generation SC superalloys have been developed by adding about 2~3 wt.% and 5~6 wt.% Ru, respectively. The sixth generation SC superalloy, TMS-238, had been developed with the composition of Co 6.5, Cr 4.6, Mo 1.1, W 4.0, Al 5.9, Ta 7.6, Re 6.4, Ru 5.0, and Hf 0.2 (wt.%)^12^, in which nine alloying elements was added and their contents count up more than 40 wt.%. Therefore, a great deal of element addition would give rise to a severe inhomogeneous composition and harmful precipitations. Thus, the means almost reaches the ultimate condition.

In the meantime, the solidification processes are also developing. The major directional solidification processes are the Bridgman technique with water cooling (WC) and liquid metal cooling (LMC)^8,13^, the downward directional solidification process (DWDS)^14^, and the zone melting liquid metal cooling (ZMLMC)^10^. These processes exhibit some disadvantage when they are industrially used to produce highly-efficient SC turbine blades, especially for large industrial gas-turbine blades. In the WC Bridgman process, the low cooling capacity induces an inhomogeneous microstructure, including the coarse dendrite, the serious microsegregation, the freckles, and the stray grains^15^. The mold used in the process is thick and nonuniform, which can generate a nonhomogeneous thermal field leading to an occurrence of stray grain^16^. The LMC Bridgman process increases the cooling capacity^13,14^. However, the coolants may contaminate the casting, which is deleterious to the mechanical properties^17^. ZMLMC is limited to the small size of the sample and in the lab. Other methods are thirstily needed to obtain high-quality castings with a homogeneous composition and structure.

An application of electromagnetic processing in solidification is an effective means that controls the material structures and further their properties^18^. The static magnetic field has maturely applied in the industrial growth of the Si semiconductor^19,20^. It has been paid little attention in controlling the directional solidification of the superalloys.

Let us review briefly the effect of the static magnetic field in solidification. The static magnetic field exhibits two effects on the melt convention and one effect in the solid during directional solidification^18–28^. One effect in the melt is the magneto-hydrodynamic damping (MHD) effect^18–21^. It has been widely used in the metal and semiconductor melt to suppress the melt turbulences and flow instabilities^18,21^. It not only improves the macrosegregation, internal cracks, and inclusions for the melt ingots but also eliminates the impurity striation, radial segregation, and microscopic inhomogeneity for semiconductor crystal. The other effect in the melt is the thermoelectromagnetic convection (TEMC) in melt during solidification^22–34^. It induces a flow in the melt and is gained by the interaction between the magnetic field and thermoelectric current owing to a thermoelectric power difference between the liquid and solid phases at the growth front. The influence of TEMC on the microstructure depends on its intensity and flow structure, which are related to the applied magnetic field intensity and direction, the temperature gradient, the solidification rate, the alloy system, and so on^22–30^. It could modify the solid-liquid interface shape^22^, the dendrite morphology^23,24^, the solute distribution^25^, the stray-crystal formation^26^, *et al*. The appropriate microstirring effect of TEMC increase the liquid-solid interface stability^27,28^ and flatness^29^ and eliminated the radial macro-segregation in the crystal^30^. The thermoelectromagnetic force (TEMF) in the solid near the liquid-solid interface can induce the dendrite irregularity and instability of the liquid-solid interface^31–33^, even the freckles, and the composition striations^33,34^.

From the above statement, each effect of magnetic fields plays the different role in the various alloy during the different solidification conditions. In our previous study, the destroying effects of TEMF on the SC integrity in nickel-based superalloy was investigated^35^, the TEMC and MHD effects on the primary dendrite-arm spacing^36,37^ and precipitation phases^37,38^ under the different parameters of solidification and magnetic fields. The present paper aims to develop and optimize the combination of the MHD, TEMC, and TEMF effects in the directional solidification of SC Nickel-based superalloys. It has been firstly found the high creep life of SC superalloys under the condition of the appropriate configuration of solidification parameters and magnetic field effect. The improvement mechanism is revealed from the macro SC integrity, the dendrite structure, the precipitation-phase morphology, the composition distribution across the multiscales from the sample to the dendrite size, and to the partition of alloying elements in γ and γ′ phases. It can be concluded that the composition homogeneity from the magnetic-field-assisted directional solidification should be responsible for the improvement of creep resistance. The work provides an effective means to obtain the high creep properties of SC nickel-based superalloys, which presents a potential application in industry. Moreover, it provides a guideline for achieving high-quality materials with multielements and multiphases using the magnetic fields.

## Results

### Creep properties

**Figure 2:**
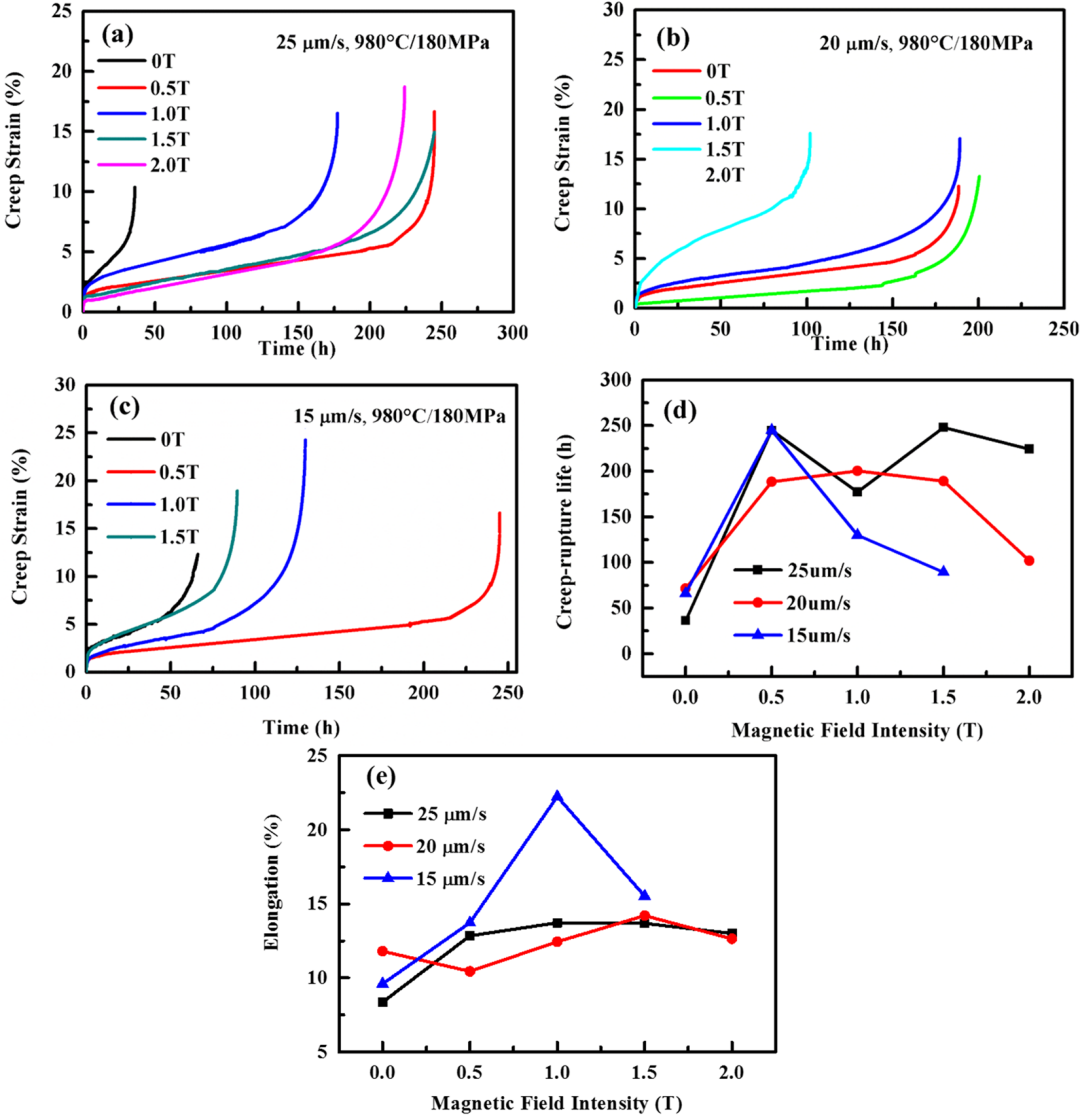
Creep behaviors of the single-crystal nickel-based superalloy at 180 MPa/980 °C. (**a**~**c**) The creep strain as a function of time with the different withdraw velocities (15, 20, and 25 μm/s) and magnetic-field intensities. (**d**) Creep life and (**e**) elongation with the magnetic-field intensity.

The creep life of the SC nickel-based superalloy directionally solidified with the different withdrawal velocities and magnetic field intensities have been studied. The creep tests have been conducted at the tensile stresses of 180 MPa and 250 MPa and at 980 °C. Figure 2(a~c) show the dependence of creep strain on time at 180 MPa/980 °C for the alloy directionally solidified at the withdrawal velocities of 25, 20, and 15 μm/s. It can be observed that the application of magnetic field during directional solidification improves the rupture life greatly. For example, the life for the alloy with 0.5 T and the drawing speeds of 15, 20, and 25 μm/s increases by 2.7, 1.6, and 5.1 times, respectively. The lifetime reaches a maximum with 0.5 T, and then decreases again. Figure 2(d) presents the relationship between the rupture time and the magnetic field intensity and shows the tendency more clearly. The elongation is also increased by the application of the magnetic field, as presented in Fig. 2(e). For example, the elongation with 0.5 T increases by 30% and 70% with 15 and 25 μm/s, respectively. The effects of the used withdrawal velocities on the superalloy creep are not concerned because they are not the main thrust of the work. The creep properties of the superalloy with different withdraw-velocities and magnetic-field intensities at the tensile stress of 250 MPa/980 °C show the same tendency as that at 180 MPa/980 °C (Fig. 1 in Supplementary). Therefore, the static-magnetic-field-assisted solidification significantly improves the creep life of the SC superalloy. In order to understand the mechanism, the microstructure features were investigated, including the composition distribution on multiple scales from the macro sample, the micro dendrite to the partition in the γ/γ′ phases, the precipitation phase, and the dendrite morphology and orientation. The fracture mode and deformation mechanism were also studied, as described below.

### Solute distribution

**Figure 3:**
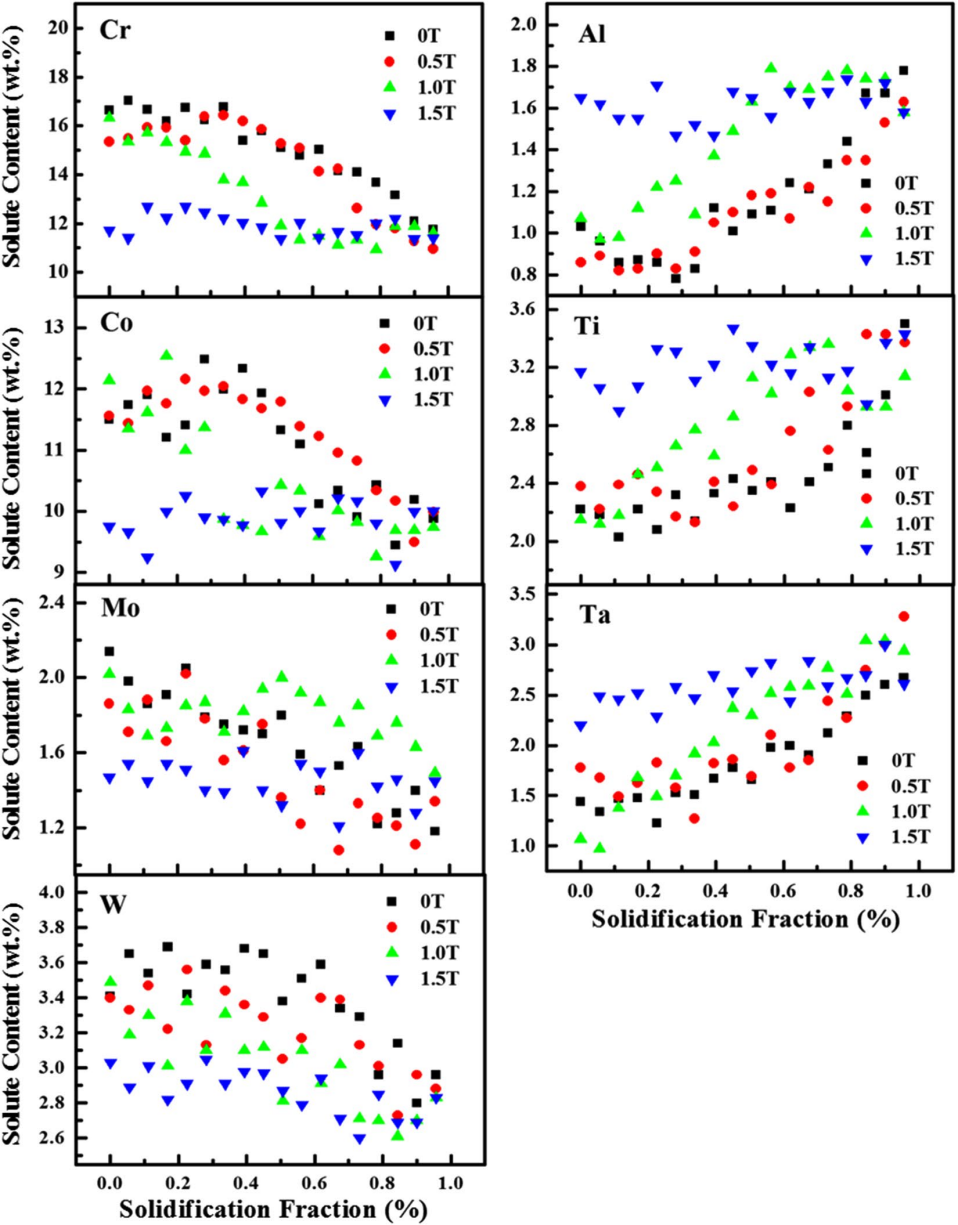
Solute distribution of alloying elements along the longitudinal section. The alloying elements are marked at the top left corner in each subgraph. The samples were solidified at 25 μm/s.

The solute distribution involves the macrosegregation on the sample scale, the microsegregation on the dendrite scale, and the partition behavior in γ and γ′ phases. The solute distribution along the longitudinal section exhibits more homogeneity in the samples solidified in the static magnetic field (Fig. 3). Especially, the 1.5 T magnetic field greatly and evenly distributes the solute along the whole sample. The solute distribution in the samples solidified in the static magnetic field and at 15 μm/s presents the same trend (Fig. 2 in Supplementary).

**Figure 4:**
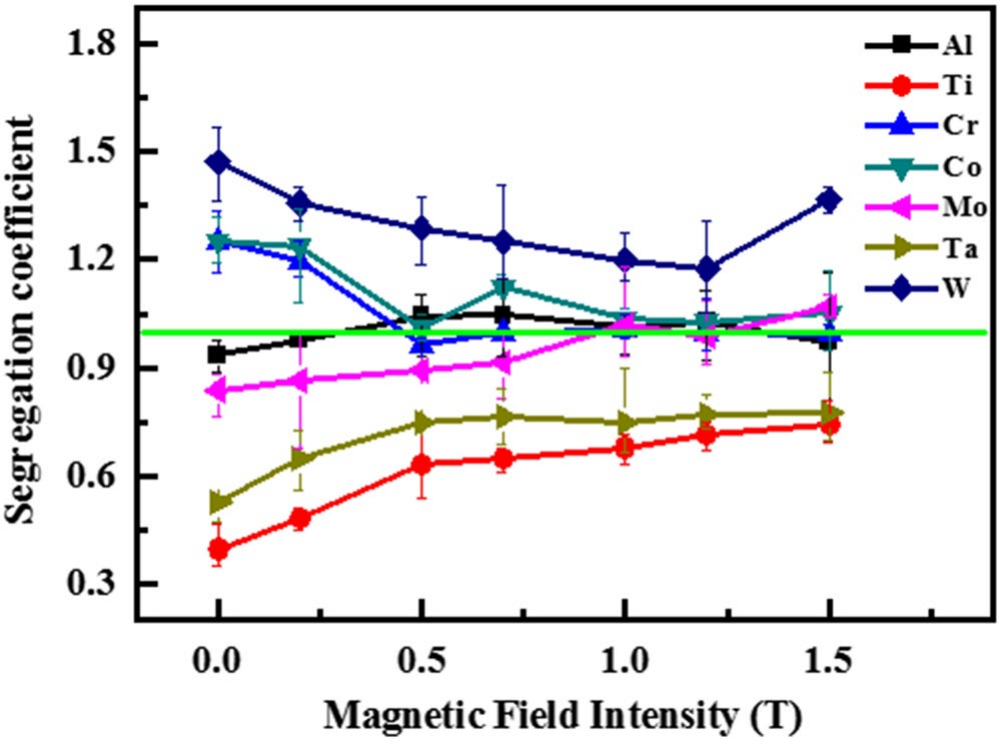
Microsegregation coefficient of alloying elements on the dendrite scale. The samples were solidified at 25 μm/s.

The micro-segregation of superalloys is evaluated by the solute distribution on a dendrite scale, which is indicated by the segregation coefficient, *k*′. *k*′ is defined as the ratio of *C*_*DC*_: *C*_*ID*_, where *C*_*DC*_ and *C*_*ID*_ denote the average concentration of each alloying element in the dendrite core and interdendrite, respectively. Al, Ti, and Ta segregate in the interdendrite, and their *k*′ values are less than one (Fig. 4), which is defined as the positive segregating elements. Cr, Co, and W segregate in the dendrite core, and their *k*′ values are more than one (Fig. 4), which is defined as the negative segregating elements. Mo segregates in the interdendrite^14^ or in the dendrite core^39^. In the study, it does from the interdendrite to the dendrite core with the increasing magnetic field. We can observe that the application of the magnetic field increases the segregation coefficients of Al, Ti, and Ta, and decreases those of Cr, Co, and W (Fig. 4). The segregation coefficient of almost each element gradually approaches one with the increased magnetic field. Therefore, the magnetic field increases the homogeneity of the solute distribution on the dendrite scale.

**Figure 5:**
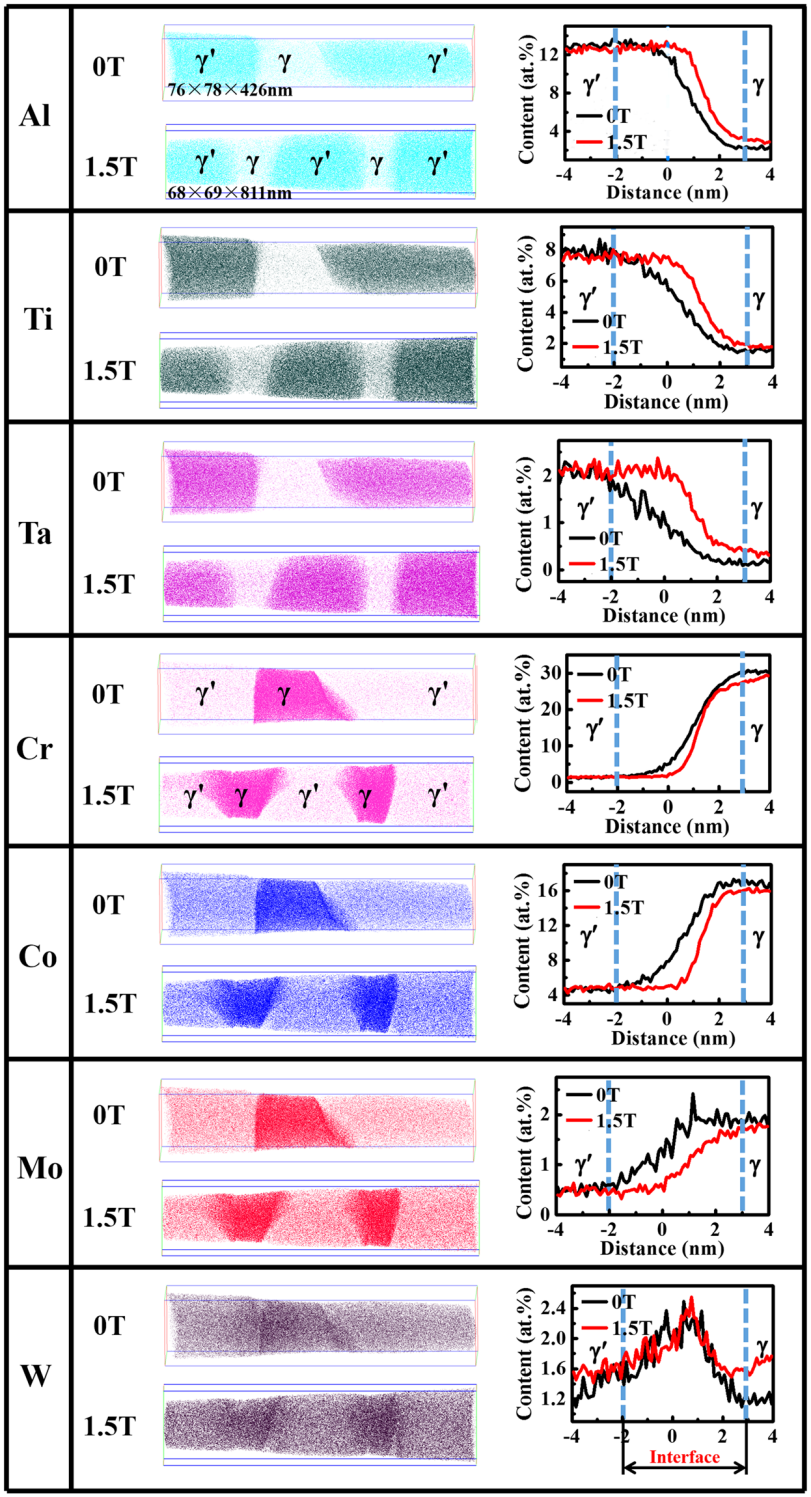
Atom-probe tomography (APT) analysis. The left-column graphs show each atom map of the atomic distribution within the γ/γ′ phase. The right-column graphs present the proximity histogram of the atom distribution across the γ/γ′ interface and within the γ/γ′ phases in the single-crystal nickel-based superalloy.

The atom-probe tomography (APT) provides further details of the elemental partition within the γ/γ′ phase. Al, Ti, and Ta are more partitioned in the γ′ phase, while Cr, Co, and Mo are more done in the γ phase (Fig. 5). W distribution within the γ and γ′ phase are almost same (Fig. 5). The application of the magnetic field generally increases the contents of Al, Ti, and Ta in the γ phase, and decreases the contents of Cr, Mo, and Co in the γ phase (Table 1 and the right-column graphs in Fig. 5). The elements in the γ′ phase are not obviously affected by the application of magnetic field except for W. Table 1 and Fig. 5 shows that the magnetic field generally decreases in the concentration difference between γ and γ′ phases. We also noticed that the interface composition across the γ/γ′ phase also changes in the samples solidified in the magnetic field, which should be consistent with the composition changes within the γ/γ′ phase and needs further investigation.

**Table 1:** The compositions within the γ/γ′ phase by APT. Note: The change extent of the elements, Δ*Cr*, is given by, ∆*C*_*r*_ = |*C*_1.5*T*_ - *C*_0*T*_ |⁄*C*_0*T*_, where *C*_*1.5T*_ and *C*_*0T*_ are the compositions at the magnetic field of 1.5 T and 0 T, respectively. The concentration difference between the γ and γ′ phases is expressed as *ΔC*, *ΔC* = |*C*_*γ*_ − *C*_*γ′*_|, where *C*_*γ*_ and *C*_*γ′*_ are the compositions in the γ and γ′ phases, respectively.

Phase	Magnetic field	Composition ratio (at.%, Ni as a balance)						
		Al	Ti	Ta	W	Cr	Co	Mo
γ	0 T	2.56	0.51	0.16	1.36	31.20	16.82	1.85
	1.5 T	2.89	0.60	0.19	1.73	29.62	15.35	1.62
	Change extent (%)	12.9	17.6	18.8	27.2	5.0	8.7	12.4
γ′	0 T	12.43	8.21	2.48	0.83	1.37	4.49	0.31
	1.5 T	12.55	8.05	2.38	1.00	1.41	4.57	0.31
	Change extent (%)	1.0	1.9	0.4	20.5	2.9	1.8	0.0
*ΔC*	0 T	9.87	7.70	2.32	0.53	29.83	12.33	1.54
	1.5 T	9.66	7.45	2.19	0.73	28.21	10.78	1.31

**Figure 6:**
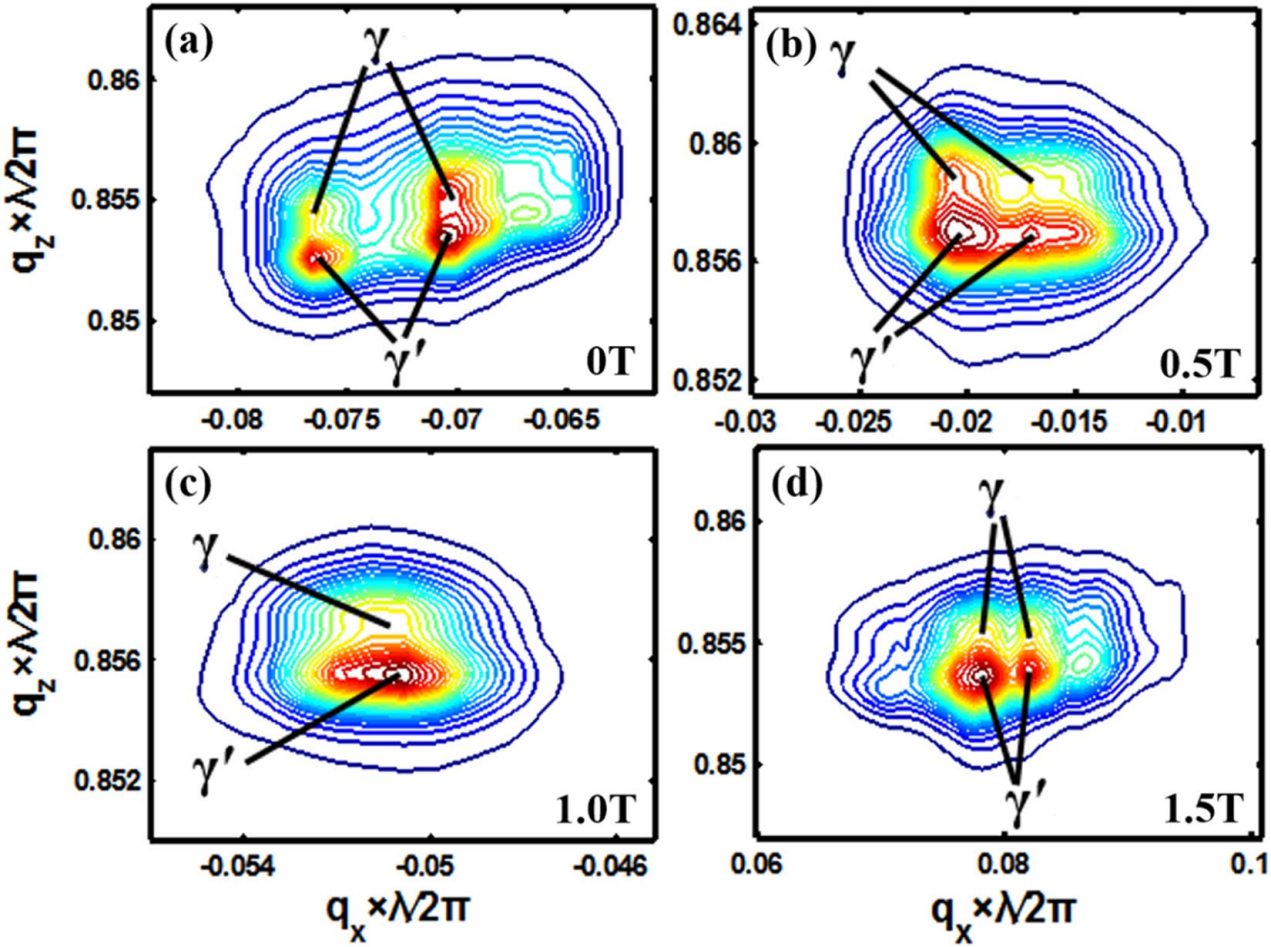
Two-dimensional iso-intensity mappings on the reciprocal space for the investigated superalloys. (**a**) 0 T. (**b**) 0.5 T. (**c**) 1 T. (**d**) 1.5 T. The line end within the iso-intensity mappings indicates the positions of γ and γ′ phases, according to which the lattice misfit was determined. The detailed procedure is stated in ref.^52^.

The change of the elemental distribution in the γ/γ′ phases further modifies the lattice misfits between the γ/γ′ phases of the alloy. The lattice misfits are 0.240%, 0.204%, 0.188%, and 0.180% in the samples prepared with 0 T, 0.5 T, 1 T, and 1.5 T, respectively (Fig. 6). The *q*_*x*_ and *q*_*z*_ in Fig. 6 are the horizontal and vertical ordinates in the reciprocal space, and are defined as Eqs (1) and (2).

1 $${q}_{x}=Rcos(2\theta -\omega )-Rcos\omega $$

2 $${q}_{z}=Rsin(2\theta -\omega )+Rsin\omega $$

where *R* = 2π*/λ* is the radius of an Ewald sphere, *λ* is the incidental wavelength, *ω* is the incident angle, and *θ* is the diffraction angle. The magnetic-field-assisted solidification decreases the lattice misfit between the γ/γ′ phase. It is consistent with the above APT results.

### Precipitation phases

**Figure 7:**
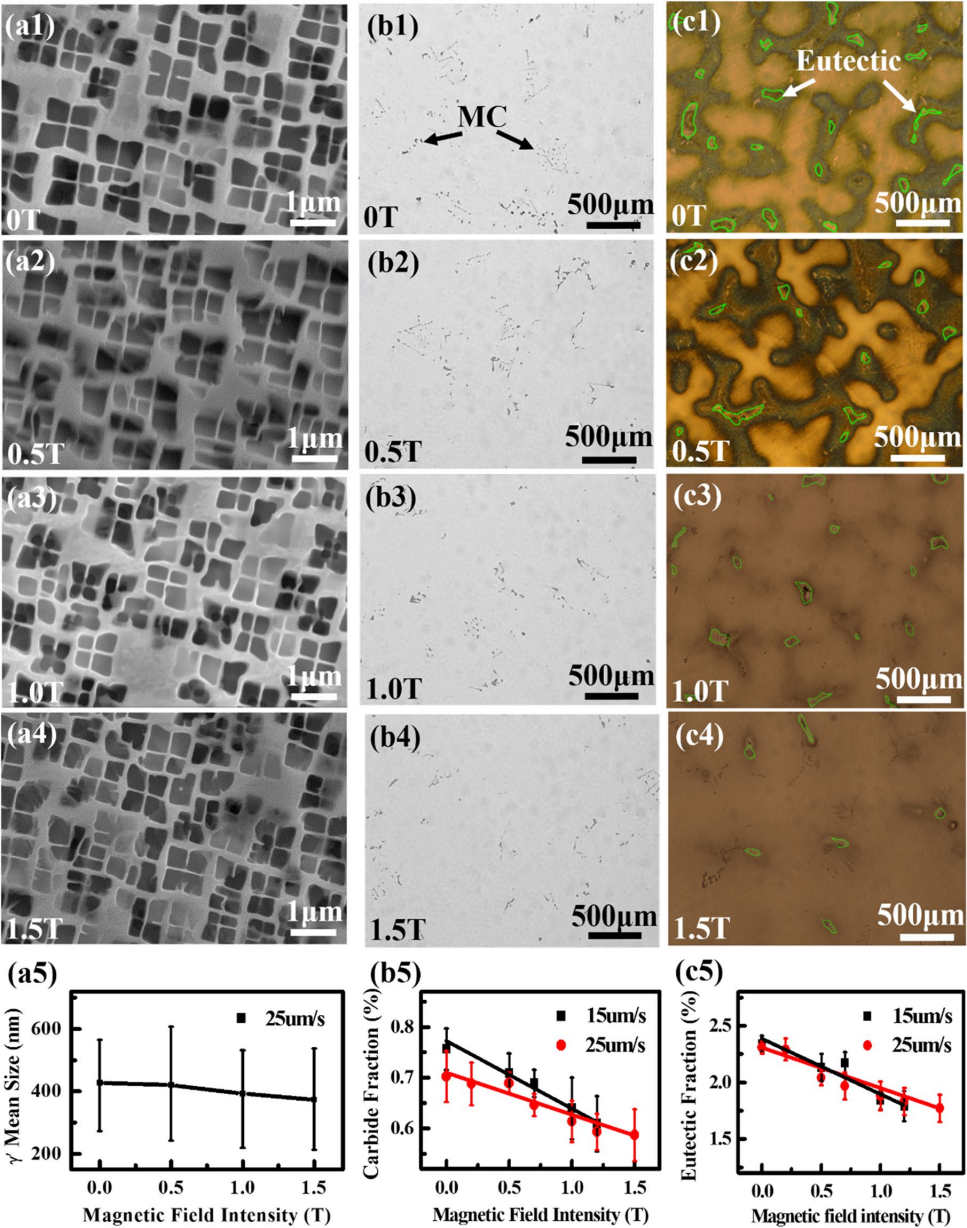
Precipitation phases in the superalloys prepared at 25 μm/s. (**a1~a5**) γ′ precipitation at the dendrite core. (**b1~b5**) carbides. (**c1~c5**) γ/γ′ eutectic phases (indicated with the green line by hand for the clear presentation).

The change of the precipitation phases is closely related to the compositional distribution. The precipitation phases of the present superalloy include the γ′ phase, MC carbide, and γ/γ′ eutectic. The magnetic field decreases the γ′ size, the contents of carbides and γ/γ′ eutectic phases (Fig. 7 and Fig. 3 in Supplementary), which is agreement with the homogeneity improvement of the solute distribution. For example, in the superalloy prepared at the withdrawal velocity of 25 μm/s, the 1.5 T magnetic field decreases the γ′ size by 12.7%, the contents of carbides and γ/γ′ eutectic phases by 16.4% and 23.4%, respectively. Moreover, the change extent of precipitation phases enhances with the increasing magnetic field intensity.

### Dendrite morphology and orientation

**Figure 8:**
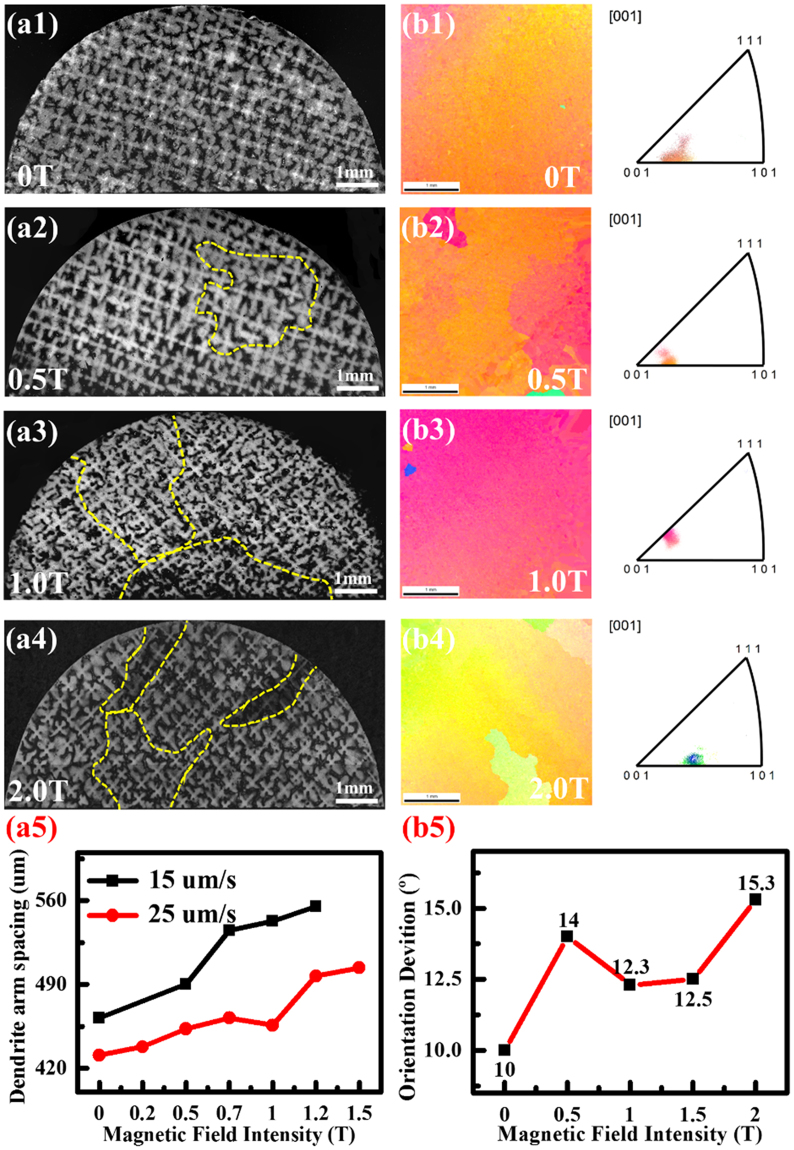
Dendrite morphology and orientation. (**a1~a4**) The dendrite morphology. (**a5**) The primary dendrite-arm spacing. (**b1~b5**) The dendrite orientation characterized by EBSD photos and the inverse pole figures. The samples were solidified at 25 μm/s. The magnetic-field-assisted solidification destroys the single-crystal integrity, as indicated by an increase in column grains (marked by the yellow dash line).

Figure 8 shows the dendrite morphology, the dendrite orientation, and the primary dendrite arm of the SC superalloy with different magnetic fields. It can be seen that the samples solidified under the magnetic fields exhibit several column grains (marked by the yellow dash-lines). It indicates that the high magnetic field breaks the integrity of the SC. It can be verified from the initial growth morphology of the dendrite, as presented in Fig. 4 in Supplementary. The stray grains form at the melted-unmelted interface of the superalloy during the initial directional solidification with the magnetic fields. They are gradually disappear under the effect of thermal flow during the directional solidification (Fig. 5 in Supplementary). It should be emphasized that the shape of liquid-solid interface at the quenched time and the morphologies of primary and second dendrites are not affected by the magnetic fields (Fig. 8 and Fig. 5 in Supplementary), which are not different from the phenomena in the references^23,24,31–33^. The primary dendrite-arm spacing is increased with the magnetic field application (Fig. 8a5). For example, the 1.5 T magnetic field increases the primary dendrite-arm spacing by about 20% compared with that of the alloy solidified in the condition of no magnetic field.

The deviation degree of the crystal orientation from the (001) growth direction also indicates the breaking effect from the magnetic field. Figure 8(b1~b5) shows the EBSD photos across the transverse section of the alloys solidified at 20 μm/s. The magnetic field increases the (001) deviation. For example, the 2 T intensity increases the deviation by 50%. Generally, the breakage of the SC integrity becomes more serious with the enhancing magnetic field intensity.

### Fracture mode and deformation mechanisms

**Figure 9:**
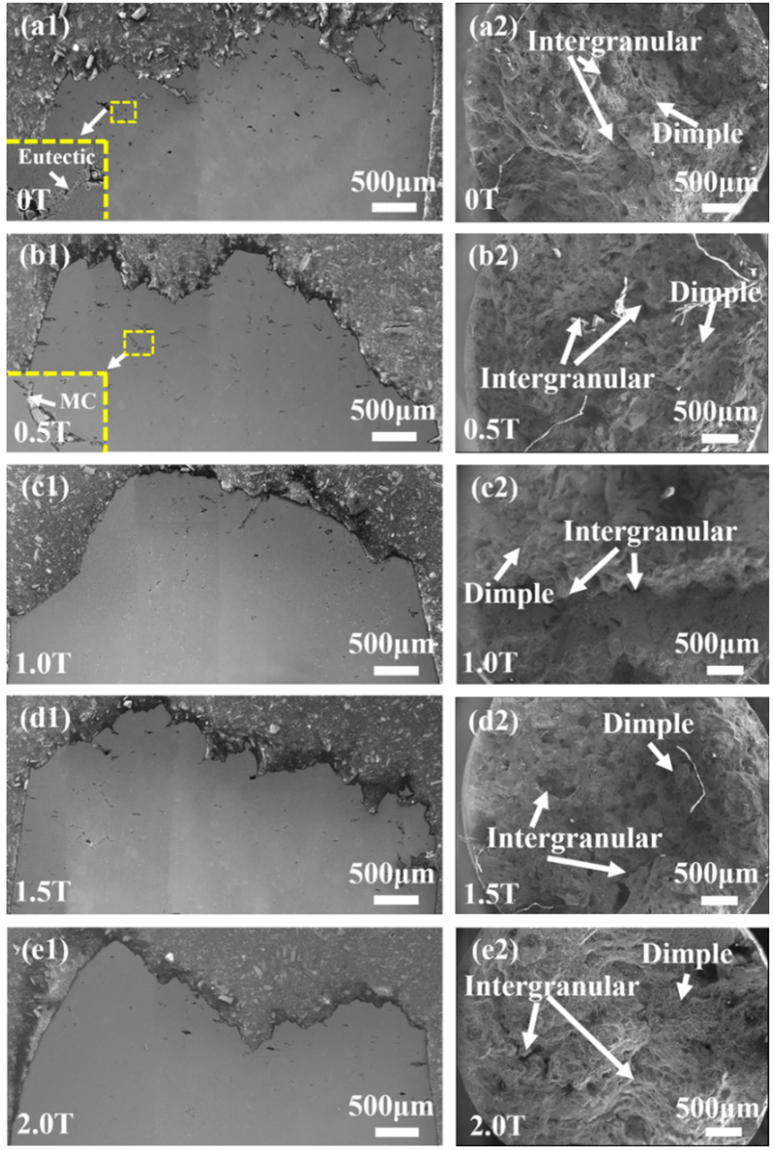
Fractography and longitudinal microstructures near fracture surface in the samples crept at 980 °C/250 MPa (solidified at 25 μm/s). (**a**) 0 T. (**b**) 0.5 T. (**c**) 1 T. (**d**) 1.5 T. (**e**) 2 T. The enlarged areas in the (**a**) and (**b**) showed that the cracks initiate around the MC carbide and eutectic phases.

**Figure 10:**
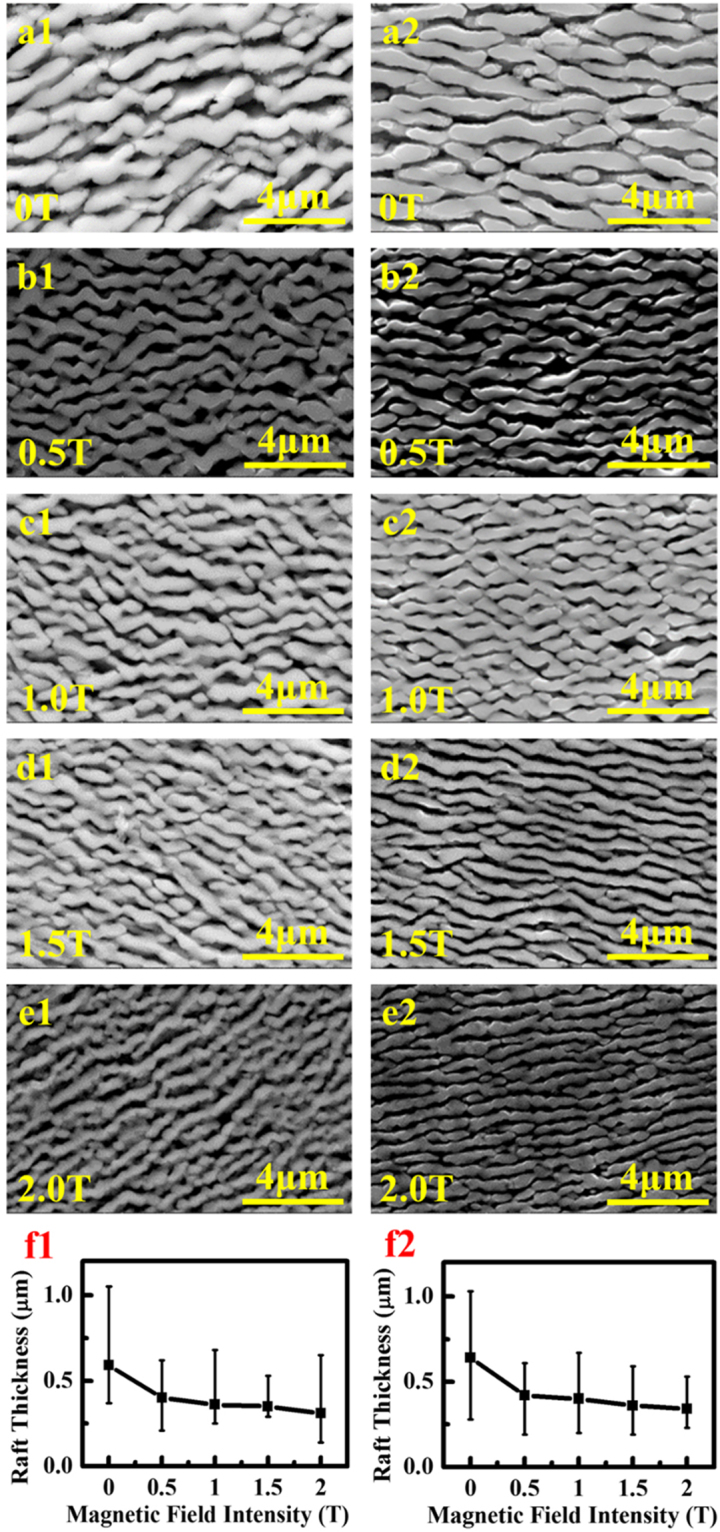
Rafting structures near fracture surface in the samples crept at 980 °C/250 MPa (solidified at 25 μm/s). (**a**) 0 T. (**b**) 0.5 T. (**c**) 1 T. (**d**) 1.5 T. (**e**) 2 T. The numbers 1 and 2 show the positions of 1 mm and 3 mm away from the fracture surface, respectively.

The fracture morphology and longitudinal microstructure near the fracture surface show that the cracks initiate around the MC carbides and eutectic phases in the interdendrite area, as indicated by the enlarged area in Fig. 9(a1) and (b1). In the meantime, the number of cracks obviously decreases in the samples prepared under the magnetic field (Fig. 6 in Supplementary). This tread is agreeable with the decreased content of the carbides and eutectic phases in the magnetic-field-assisted samples. The rafting thickness of the longitudinal microstructure decreases with the increasing magnetic field (Fig. 10), which accords well with the change of γ′ size with magnetic field. All the specimens show the mixed fracture feature of the interdendrite and dimples (Fig. 9a2~e2). The magnetic-field-assisted solidification increases the portion of the dimple fraction, which can be confirmed by the increase in the fluctuation degree of the fracture profile (Fig. 9a1~e1).

**Figure 11:**
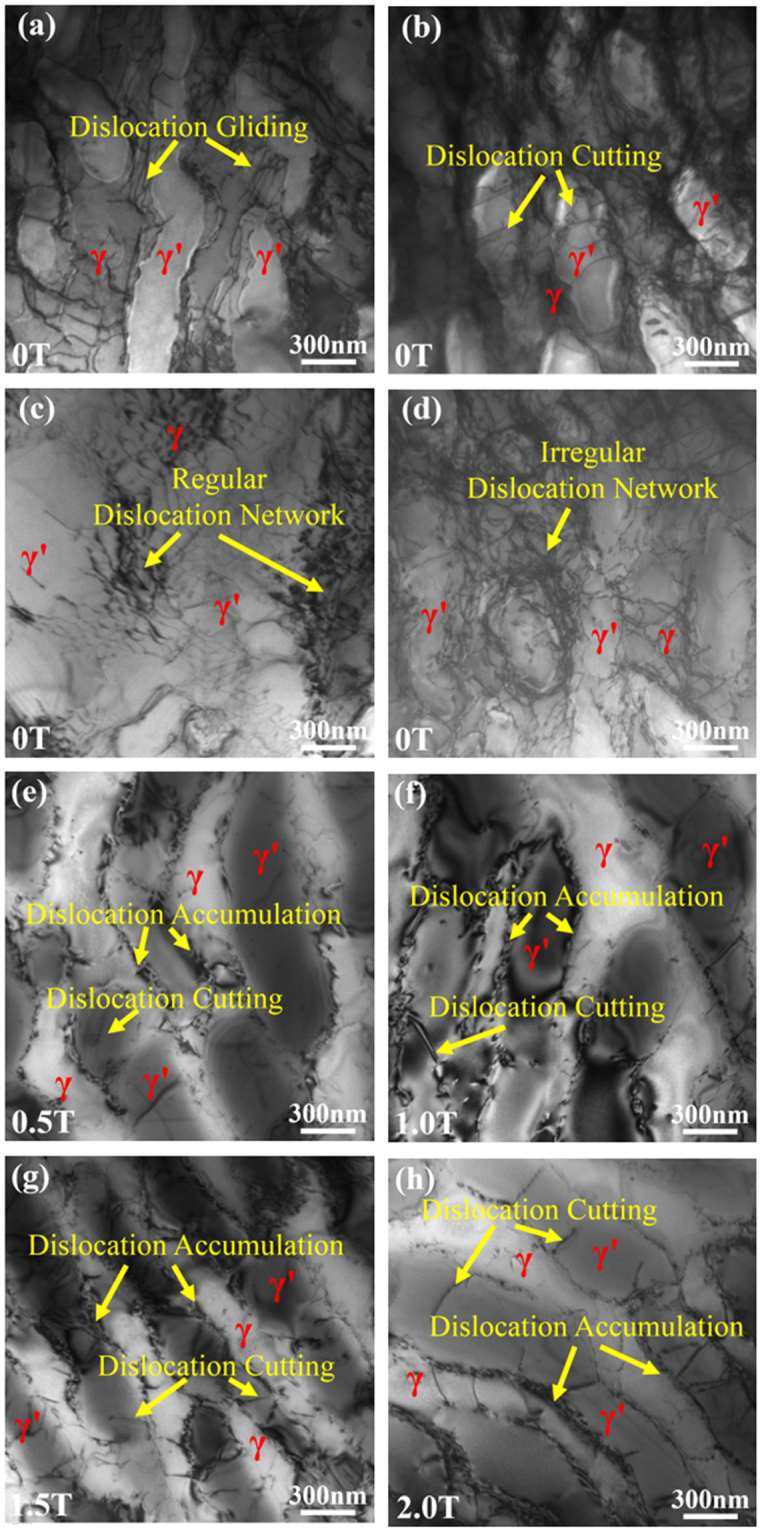
Dislocation characteristic in the samples crept at 980 °C/250 MPa (solidified at 25 μm/s). In the case of 0 T, there is dislocation gliding in the γ channel (**a**), dislocation cutting in the γ′ phase (**b**), and regular (**c**) and irregular (**d**) dislocation network at the γ/γ′ interface and in the γ channel. In the samples prepared under 0.5 T (**e**), 1 T (**f**), 1.5 T (**g**), and 2 T (**h**), the dislocation accumulated at the γ/γ′ interface and cut in the γ′ phase.

The creep deformation is accompanied by the dislocation motion. The main characteristic of dislocation motion in the SC nickel-based superalloy crept under the high temperatures and low stresses is its accumulation in the γ matrix channels, the network formation at the γ/γ′ interface, and cutting the γ′ phase^40–42^. The accumulation of the different dislocation could promote the formation the network formation at the γ/γ′ interface. The present creep was conducted in the high temperatures and low stresses^43,44^. The dislocations glide is observed in the γ channel of the samples without the magnetic field (Fig. 11a) and does not appear in the samples with the magnetic fields (Fig. 11e~h). A number of dislocation networks forms at the γ/γ′ interface and in the γ channel of the samples without the magnetic field (Fig. 11c and d) and is not observed in the samples with the magnetic fields (Fig. 11e~h). The dislocation accumulates at the γ/γ′ interface in the samples with the magnetic field (Fig. 11e~h). The dislocation quantity in the γ channel of the samples without the magnetic field is much higher than that in the samples with the magnetic field by observing the many various fields under the transmission electron microscope (TEM). The dislocations cut in the γ′ phase in the samples both without (Fig. 11b) and with the magnetic fields (Fig. 11e~h). But the number of cutting dislocations in the samples without the magnetic field is less than that with the magnetic fields. Therefore, from the dislocation type, it can be concluded that the samples solidified with the magnetic fields have the same crept mechanism as that without the magnetic fields. From the dislocation quantity in the γ and γ′ phases, the samples with the magnetic fields show the greater resistance to the creep deformation.

## Discussions

The above results shows that the static-magnetic-field-assisted solidification significantly improves the creep properties of the SC superalloy. And it also affects the structures from the macro to micro scales, which is embodied in the increase in the composition homogeneity on the multiscales, the decrease in the γ′ phase size and the contents of the carbides and eutectic phases, the breakage in the SC integrity, and the increases in the primary dendrite-arm spacing, as presented in Table 2. Except for the SC integrity and dendrite-arm spacing, the changes of other structures benefit the improvement of creep properties.

**Table 2:** Changes of structures across the multiple scales in the magnetic-field-assisted solidification, their benefit for creep properties, and from a certain effect of the magnetic field.

Structures across multiple scales	Changes in magnetic-field-assisted solidification	Benefits for creep property	From what effect of magnetic field
Single-crystal integrity	Destroy	No	TEMF
Primary dendrite-arm spacing	Increase	No	MHD
Macrosegregation	Decrease	Yes	MHD
Microsegragation	Decrease	Yes	MHD
Element partition difference in γ/γ′ phases	Decrease	Yes	Related to macro- and micro-segregation
Lattice misfit of γ/γ′ phases	Decrease	Yes	Related to macro- and micro-segregation
γ′ size, carbide and eutectic contents	Decrease	Yes	Related to macro- and micro-segregation

As stated in the introduction, the static magnetic field exhibits two effects (MHD and TEMC) on the melt convention and one effect (TEMF) in the just-frozen solid in directional solidification^18–30^. The effect on the melt convection mainly involves two aspects: MHD and TEMC. The MHD effect plays a damping role in the melt, such as motion braking, convection stabilization, and free-surface shaping, which can affect the macrosegregation and inclusions for the melt ingots^18^, the impurity striation and microscopic uniformity for the semiconductor crystal^19–21^. The TEMC stems from the interaction of the magnetic field and the thermoelectric current at the liquid-solid interface and its affecting zone is near the interface^22,23,27^. It plays a stirring role at the interface’s front in the radial direction. The appropriate magnitude of TEMC results in a stability in the liquid-solid interface^27^. However, the strong TEMC could modify the liquid-solid interface shape and the dendrite morphology, or induce the macrosegregation^22–24,45,46^. The two effects compete. Khine *et al*. showed that the magnitude of the meridional circulation first increased from zero to a maximum and then decreased, because the MHD effect increased faster than the TEMC effect^23,30^. When Hartmann number, Ha, equals to approximately 10, the TEMC effect reaches the maximum condition^30^. Ha is defined as,

3 $$Ha=BL\sqrt{\frac{\sigma }{v}}$$

where *B* is the magnetic field intensity, *L* is the characteristic length of the melt, *σ* is the electrical conductivity of the melt, and *ν* is the kinematic viscosity of the melt. In the present work, Ha is estimated to be 188 in the case of B = 0.5 T, L = 400 μm, *σ* = 1.3 × 10^6^ Ω^−1^ m^−1^ ^47^, and *ν* = 0.75 × 10^–6^ m^2^ s^−1^ ^48^. Therefore, the MHD effect dominates the melt convection in the work, which could be demonstrated by the increase of temperature gradient in the melt with magnetic field (Fig. 7 in Supplementary).

**Figure 12:**
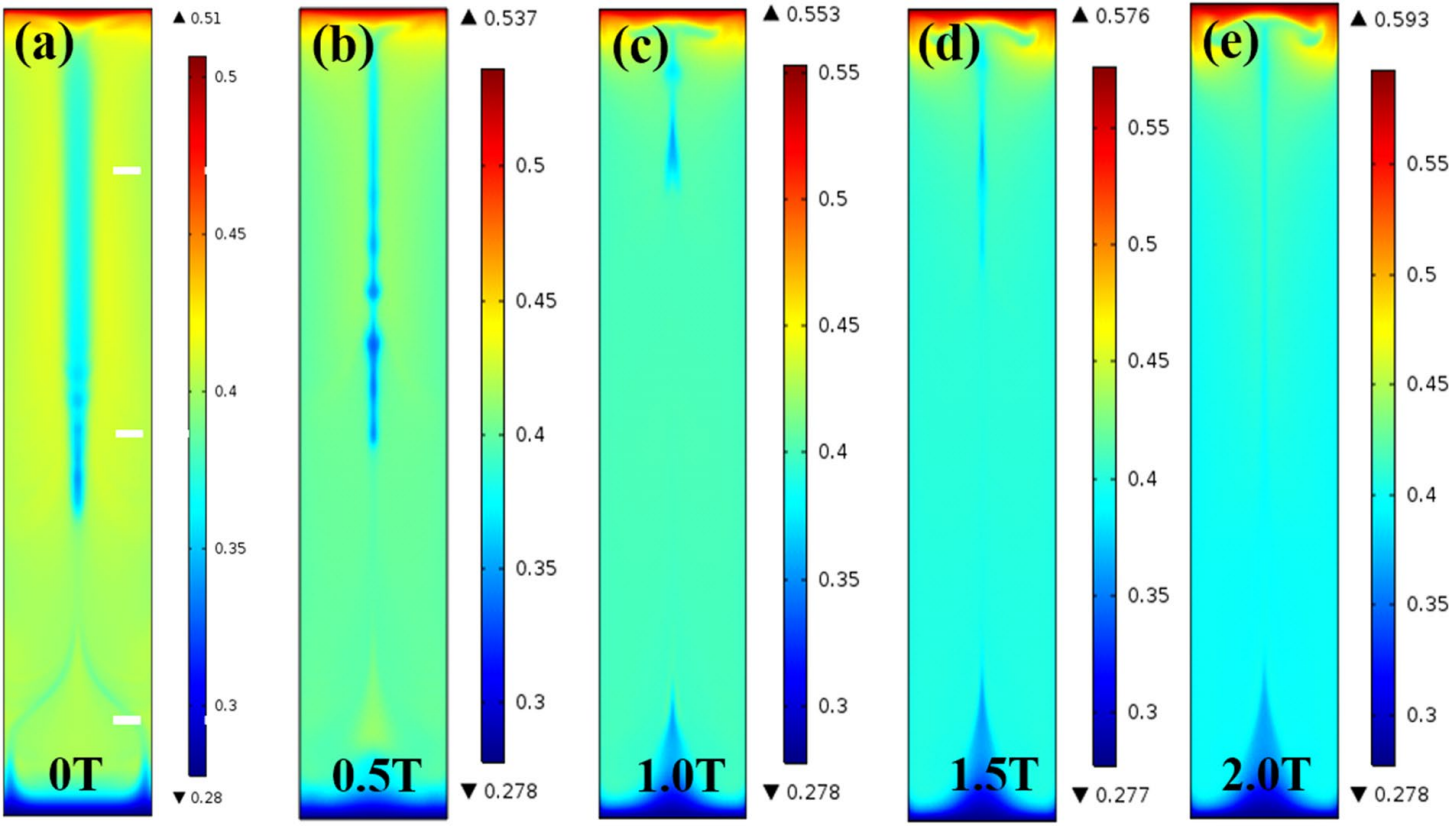
The effect of the magnetic field on the solute distribution in the Ni-40.38 wt.% Cu alloy. (**a**) 0 T. (**b**) 0.5 T. (**c**) 1 T. (**d**) 1.5 T. (**e**) 2 T.

The effect of MHD on the solute distribution in the Ni-40.38 wt.% Cu was investigated by the numerical simulation in the present study. The solute distribution on the macro-sample scale is more homogeneous with the MHD effect (Fig. 12), which is achieved through its influence on the convection and temperature fields. The schematic diagram of the simulation growth configuration, the applied parameters and the convection field, and the temperature field are shown in Figs 8~12 and Table 1 in Supplementary. Therefore, the MHD effect in the whole melt should be responsible for the homogeneous distribution on the macro scale, which could be verified by the increase in the temperature gradient in the melt (Fig. 12 in Supplementary). The simulated changes of temperature gradient is consistent with the experimental ones.

The microsegregation of alloying elements is determined by the partition behavior in the liquid and solid phases near the interface. It is evaluated by the effective distribution coefficient *k*_*e*_,

4 $${k}_{e}=\frac{{k}_{0}}{{k}_{0}+(1-{k}_{0})exp(-\frac{R}{D}\delta )}$$

where *k*_0_ is the equilibrium partition coefficient, *R* is the solidification rate, *D* is the diffusion coefficient, and *δ* is the thickness of solute-enrichment layer. From above the discussion, the MHD effect plays the main role in the melt in the present work. It would result in an increase in *δ*. The magnetic field intensity in this study was not sufficiently large to affect the *k*_0_ and *D*^27,31^. Therefore, the *δ* increase brings about the enhancement of *k*_*e*_, which decreases the microsegregation of alloying elements.

The change of precipitation phases is closely related with the micro- and macro-segregation of alloying elements. The increase in the homogeneous extent of the alloying elements would result in an increase in the γ′ phase content and a decrease in the MC-carbide and eutectic contents. The γ′-phase precipitates from the γ matrix after the liquid phase completely solidifies. The quantity of the solute elements is more in the γ matrix with the magnetic field than that without the magnetic field during initial solidification because of the decreased segregation, which maybe be related to the reduction of the partition difference of elements between the γ/γ′ phases.

According to Kurz-Fisher model, the primary dendrite-arm spacing *λ*_1_ is expressed as^49^,

5 $${\lambda }_{1}=4.3{(\frac{{\rm{\Delta }}{T}_{0}D\Gamma }{{k}_{0}})}^{\frac{1}{4}}{G}^{-\frac{1}{2}}{R}^{-\frac{1}{4}}$$

where *ΔT*_0_ is the solidification interval, *Γ* is the Gibbs-Thomson coefficient, *G* is the temperature gradient, and other symbols are same as the above. As stated above, the magnetic field intensity in this study was not sufficiently large to affect the *k*_0_, *Γ*, and *D*. The effect of magnetic field on *G* has been studied (Fig. 7 in Supplementary), but the one on *ΔT*_0_ and *R* has not reported up to now to our knowledge, which need further investigation. However, *λ*_1_ shows an increase when MHD effect dominates the melt convection^36,37^, which is consistent with the present results and the above discussion.

**Figure 13:**
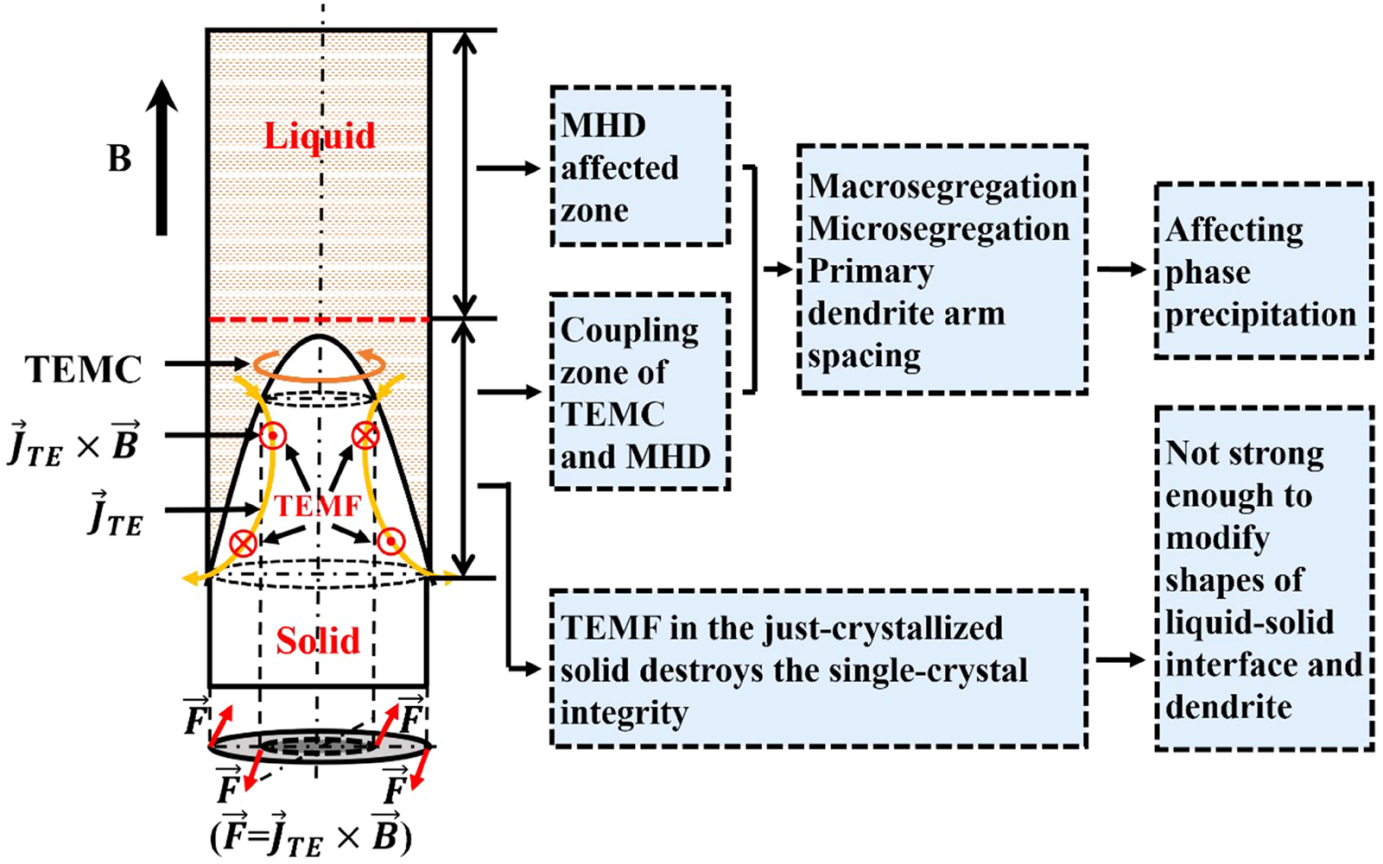
The illustration for the affected zone of the multiple effects from the static magnetic field and their influence on the structure and solute distribution. It should be emphasized that the TEMF in the just-crystalized solid are not strong enough to modify the liquid-solid interface shape and the dendrite morphologies in the present study.

The TEMF effect in the just-frozen solid during solidification can induce the irregularity of the dendrite and the instability of the liquid-solid interface^31,32^, even the freckles and striations^33,34^. Accordingly, the breakage on the single-crystal perfection with the magnetic fields should result from the TEMF effect. The TEMF in the present study is not strong enough to modify the liquid-solid interface shape and the dendrite morphology, which greatly changes in the reports^26,31,32^. The TEMF is given by,

6 $${F}_{TEMF}=\frac{-{\sigma }_{L}{\sigma }_{s}{f}_{L}}{{\sigma }_{L}{f}_{L}+{\sigma }_{s}{f}_{s}}({S}_{s}-{S}_{L})\nabla TB$$

where *σ* is the electrical conductivity, *f* is the fraction, *S* is the thermoelectric power, $\nabla$*T* is the temperature gradient, *B* is the magnetic field intensity, the lower right letters of *L* and *s* represent the liquid and solid, respectively. Equation (6) shows that the TEMF in the solid increases linearly with the rising magnetic field, which well explains that the breakage of the single-crystal perfection increases with the magnetic fields. The breakage effect offsets the role of the composition homogeneity and the precipitation phases for the creep properties at the higher magnetic field. Therefore, the creep lifetime goes through the maximum with the magnetic fields, i.e. reaches a maximum with 0.5 T and then decreases again, in the present work. The above discussion about the multiple effects of the magnetic field on the structure aspects of the superalloy are also summarized in Table 2 and Fig. 13.

The change of the composition distribution, the precipitation phases, and the element partition between on the γ/γ′ phases from the magnetic-field-assisted solidification could be verified from the crack number, the raft thickness, the fracture mode, and the dislocation number in the crept samples. The decreases in the content of carbide and γ/γ′ eutectic and the γ′ size correspond to the reduction in the crack number and the raft thickness in the crept structure, respectively. The application of the magnetic field generally increases the contents of Al, Ti, Ta, and W and decreases the contents of Cr, Mo, and Co in the γ phase. The solid-solute strengthening effects of Al, Ti, Ta, and W are more dramatic than those of Cr, Mo, and Co in the γ phase^50^. The W content in the γ′ phase is increased dramatically, and the other elements have no obvious change. Therefore, the variations of the element partition increase the strengthes of the γ and γ′ phases, which increase their creep resistance and decrease the dislocation quantity during the crept deformation. The decrease in the lattice misfits between the γ/γ′ phases also benefit the creep improvement since it corresponds to the small interface energy and obviously suppresses the coalescence of γ′ phase^51^. All the changes in the structure increase the strength and the ductile fracture of the superalloy, and finally improve the creep life.

### Conclusions

Our study reveals that the magnetic-field-assisted directional solidification greatly improves the creep life of a SC nickel-based superalloy. The improvement is achieved by a balance of the structure change from macro to micro scales. The magnetic-field-assisted directional solidification increases the homogeneous extent of solute distribution across multiple scales and decreases the γ′ size, the contents of carbide and eutectic phases, which are helpful for the improvement of the creep properties. The SC integrity is broken and the primary dendrite-arm spacing is lightly increased, which does not benefit the creep resistance. The morphologies of liquid-solid interface and dendrite are not affected. The changes of the structure and the precipitate phases on the multiple scales arise from the multiple effects of the magnetic field.

The crack number and the rafting thickness in the longitudinal deformed structure obviously decreases in the samples prepared under the magnetic field. It decreases with increasing the magnetic field intensity. This tread is agreement with the decreased content of the carbide and eutectic phases and the change of γ′ size in the samples solidified with the magnetic field. The dislocation quantity in the γ and γ′ phases in the samples solidified with the magnetic fielsd is much less than that without the magnetic field, which is consistent with the change of element partition.

## Experimental Methods

### Sample preparation

The material used in the present work was the first generation SC Ni-based superalloy, PWA1483, applied in industrial gas turbines. There was no specific reason using the material because the work mainly aimed at clarifying the effect of magnetic field on the creep and microstructure of the SC superalloy. The chemical compositions of the alloy were C 0.067, Al 3.58, Ti 4.07, Ta 4.99, Cr 12.03, Co 8.94, Mo 1.84, W 3.91 (wt.%), and Ni as a balance. The alloy ingot was prepared by inducting melting. The SC samples were obtained by seed crystals. The directional solidification was conducted with the conventional Bridgman liquid-metal-cooling technique in an argon environment. The experimental apparatus consisted of a static superconductor magnet, a Bridgman-Stockbarge type furnace, a drawing velocity and temperature controller. The apparatus was adjusted to make sure that the liquid-solid interface was in the center plane of the magnet where the magnetic-field intensity was static. The SC specimen with a diameter of 9 mm and a length of 80 mm was obtained. The withdrawal speeds of 15, 20, and 25 μm/s and the magnetic field intensities of 0, 0.5, 1, 1.5, and 2 T were applied. The three withdrawal velocities with the small gap did not obviously affect the mechanical properties of the SC superalloy and were used in order to well reveal the magnetic field effects for the more experimental dada.

### Creep property tests

The ingots were given the standard heat treatment as follows: 1,230 °C/2 h/AC + 1,080 °C/2 h/AC + 850 °C/24 h/AC. Creep rupture tests were performed to fracture at 980 °C/250 MPa and 980 °C/180 MPa in air. The creep specimens were 5 mm in diameter and 25 mm in gauge length. The temperature along the gauge length was maintained at ±1 °C throughout the creep test.

### Microstructure characterization

TEM, APT, scanning electron microscope (SEM), electron-backscatter diffraction (EBSD), and energy disperse spectroscopy (EDS) were used to observe and analyze the microstructures and compositions. The transverse section was cut from the solid zone (3 cm away from the solid line) of the SC specimen to be analyzed on their phases, macrostructures, dendrite orientations, microsegregation, and element partition and lattice misfit between γ and γ′ phases. The γ/γ′ eutectic and carbides phases were examined by optical microscopy (OM) in the chemically-etched and unetched conditions, respectively. The chemical etchant was CuSO_4_ (4 g): HCl (20 ml): H_2_SO_4_ (12 ml): H_2_O (25 ml). The sizes and volume fractions of the precipitated phases were determined by the image analysis software (SISC-IAS) based on more than ten images at different areas. The compositional distribution on the macro sample from the whole longitudinal section was examined in the area scanned by the X-ray EDS. Each area was 0.3 mm (longitudinal direction) × 3.0 mm (transverse direction). The 23 areas were analyzed along the longitudinal section from the initial melt to the final solidification. The microsegregation of the alloying element was determined by EDS. Each point was averaged on the five dendrites located at the different areas. The lattice misfit of γ/γ′ phases was investigated by the two-dimensional X-ray diffraction at room temperature. The detailed procedure was stated in ref.^52^. The crystal orientation across the transverse direction of samples was investigated by the EBSD technology in an SEM (Apollo 300, Obducat CamScan Ltd., Cambridge, UK) equipped with the Channel 5 analysis software (Oxford instruments, Oxford, UK). The element-partitioning behavior between the γ and γ′ phases was analyzed by APT. The APT specimens were cut into the small rod with a diameter of 0.5 mm and a length of 15 mm. The needle samples for the APT analysis were prepared by standard two-step electro-polishing procedures. Then it was analyzed, using a CAMECA 3000 h local electrode atom probe (LEAP). The crept-fracture surface were observed by SEM (Apollo 300). The crept microstructures were investigated in TEM (JEM-2100F). The TEM foils were cut from the gauge section parallel to the longitudinal axis and mechanically ground down to approximately 50 μm. The ground foils were thinned by the twin jet polishing using an electrolyte consisting of 95 volume percent (vol.%) ethanol and 5 vol.% perchloric acid at 243 K and 30 V.

### Numerical simulation

The effect of MHD on the composition field, temperature distribution, and fluid field was simulated by the finite elements method. The detailed was described in the Supplementary Materials.

## Electronic supplementary material

Supplementary materials

## References

[1] Mughrabi H (2009). Microstructural aspects of high temperature deformation of monocrystalline nickel base superalloys: some open problems. Mater. Sci. Technol..

[2] Van Sluytman JS, Pollock TM (2012). Optimal precipitate shapes in Nickel-base γ–γ′ alloys. Acta Mater..

[3] Vogel F (2013). Mapping the evolution of hierarchical microstructures in a Ni-based superalloy. Nat. Commun..

[4] Ma DX (2016). Freckle formation during directional solidification of complex castings of superalloys. Acta Metal. Sinica.

[5] Beckermann C, Gu JP, Boettinger WJ (2000). Development of a freckle predictor via rayleigh number method for single-crystal Nickel-base superalloy castings. Metal. Mater. Trans. A.

[6] Franke MM, Hilbinger RM, Lohmüller A, Singer RF (2013). The effect of liquid metal cooling on thermal gradients in directional solidification of superalloys: thermal analysis. J. Mater. Proc. Technol..

[7] Liang YJ, Li A, Cheng X, Pang XT, Wang HM (2016). Prediction of primary dendritic arm spacing during laser rapid directional solidification of single-crystal Nickel-base superalloys. J. Alloys Compd..

[8] Nathal MV (1987). Effect of initial gamma prime size on the elevated temperature creep properties of single crystal Nickel base superalloys. Metal. Mater. Trans. A.

[9] Pyczak F, Devrient B, Neuner FC, Mughrabi H (2005). The influence of different alloying elements on the development of the γ/γ′ microstructure of Nickel-base superalloys during high-temperature annealing and deformation. Acta Mater..

[10] Liu L, Huang TW, Zhang J, Fu HZ (2007). Microstructure and stress rupture properties of single crystal superalloy CMSX-2 under high thermal gradient directional solidification. Mater. Lett..

[11] Fuchs GE (2001). Solution heat treatment response of a third generation single crystal Ni-base superalloy. Mater. Sci. Eng. A.

[12] Yuan Y (2014). Creep deformation of a sixth generation Ni-base single crystal superalloy at 800 °C. Mater. Sci. Eng. A.

[13] Brundidge CL, Drasek DV, Wang B, Pollock TM (2012). Structure refinement by a liquid metal cooling solidification process for single-crystal nickel-base superalloys. Metal. Mater. Trans. A.

[14] Wang F, Ma D, Bogner S, Bührig-Polaczek A (2015). Comparative study of the segregation behavior and crystallographic orientation in a Nickel-based single-crystal superalloy. J. Alloys Compd..

[15] Gao SF (2012). Grain selection during casting Ni-base single-crystal superalloys with spiral grain selector. Metal. Mater. Trans. A.

[16] Ma D, Lu H, Bührigpolaczek A (2012). Experimental trials of the thin shell casting technology for directional solidification. IOP Conference Series Mater. Sci. Eng..

[17] Holt RT, Wallace W (1976). Impurities and trace elements in Nickel-base superalloys. Int. Mater. Rev..

[18] Li BQ (1998). Solidification processing of materials in magnetic fields. JOM.

[19] Series RW, Hurle DTJ (1991). The use of magnetic fields in semiconductor crystal growth. J. Cryst. Growth.

[20] Hirata H, Hoshikawa K (1989). Silicon crystal growth in a cusp magnetic field. J. Cryst. Growth.

[21] Yasuda H, Toh T, Iwai K, Morita K (2007). Recent progress of EPM in steelmaking, casting, and solidification processing. ISIJ Inter..

[22] Wang J (2016). Thermoelectric magnetohydrodynamic flows and their induced change of solid-liquid interface shape in static magnetic field-assisted directional solidification. Metal. Mater. Trans. A.

[23] Li X, Gagnoud A, Ren ZM, Fautrelle Y, Moreau R (2009). Investigation of thermoelectric magnetic convection and its effect on solidification structure during directional solidification under a low axial magnetic field. Acta Mater..

[24] Zhong H (2016). Effect of interdendritic thermoelectric magnetic convection on evolution of tertiary dendrite during directional solidification. J. Cryst. Growth.

[25] Zhong H (2016). Effect of a high static magnetic field on microsegregation of directionally solidified Al-4.5Cu alloy. Acta Metal. Sinica.

[26] Zhong H, Li CJ, Wang J, Ren ZM, Zhong YB (2016). Effect of a high static magnetic field on the origin of stray grains during directional solidification. Mater. Trans..

[27] Ren WL (2016). Non-monotonic changes in critical solidification rates for stability of liquid-solid interfaces with static magnetic fields. Scientific Reports.

[28] Li M (2011). Effect of high longitudinal magnetic field on interface stability and morphology of directionally solidified Al-0.85%Cu alloy. Chinese J. Nonferrous. Metals.

[29] Yesilyurt S, Vujisic L, Motakef S, Szofran FR, Volz MP (1998). A numerical investigation of the effect of thermoelectromagnetic convection (TEMC) on the bridgman growth of Ge_1−x_Si_x_. J. Cryst. Growth.

[30] Khine YY, Walker JS (1998). Thermoelectric magnetohydrodynamic effects during Bridgman semiconductor crystal growth with a uniform axial magnetic field: large hartmann-number asymptotic solution. J. Cryst. Growth.

[31] Li X (2009). Effect of a high magnetic field on the morphological instability and irregularity of the interface of a binary alloy during directional solidification. Acta Mater..

[32] Wang J (2012). Thermoelectric magnetic force acting on the solid during directional solidification under a static magnetic field. Appl. Phys. Lett..

[33] Lehmann P, Moreau R, Camel D, Bolcato R (1998). Modification of interdendritic convection in directional solidification by a uniform magnetic field. Acta Mater..

[34] Dold P, Cröll A, Benz KW (1998). Floating-zone growth of silicon in magnetic fields. I weak static axial fields. J. Cryst. Growth.

[35] Hu ZN (2013). Effect of longitudinal static magnetic field on microstructure and creep property in single crystal superalloy DD483. Shanghai. Metals.

[36] Zhang T (2009). Effect of high magnetic field on the primary dendrite arm spacing and segregation of directionally solidified superalloy DZ417G. J. Alloys Compd..

[37] Xuan WD (2017). Effects of a high magnetic field on the microstructure of Ni-based single-crystal superalloys during directional solidification. Metal. Mater. Trans. A.

[38] Ren WL (2013). The effect of magnetic field on precipitation phases of single-crystal nickel-base superalloy during directional solidification. Mater. Lett..

[39] Wilson BC, Culter ER, Fuchs GE (2008). Effect of solidification parameters on the microstructures and properties of CMSX-10. Mater. Sci. Eng. A.

[40] Srinivasan R, Eggeler GF, Mills MJ (2000). Gamma prime-cutting as rate-controlling recovery process during high-temperature and low-stress creep of superalloy single crystals. Acta Mater..

[41] Kostka A (2007). L_12_-phase cutting during high temperature and low stress creep of a Re-containing Ni-base single crystal superalloy. J. Mater. Sci..

[42] Zhang JX, Harada H, Koizumi Y (2006). New configuration of a [001] superdislocation formed during high-temperature creep in the γ′ phase of a single-crystal superalloy TMS-138. J. Mater. Res..

[43] Nie JF, Liu ZL, Liu XM, Zhang Z (2009). Size effects of γ′ precipitates on the creep properties of directionally solidified nickel-base superalloys at middle temperature. Comput. Mater. Sci..

[44] Wang XG (2015). Creep deformation related to dislocations cutting the γ′ phase of a Ni-base single-crystal superalloy. Mater. Sci. Eng. A.

[45] Hu SD (2017). Effect of a magnetic field on macro segregation of the primary silicon phase in hypereutectic Al-Si alloy during directional solidification. J. Alloys Compd..

[46] Li X, Fautrelle Y, Ren ZM, Moreau R (2017). Formation mechanism of axial macrosegregation of primary phases induced by a static magnetic field during directional solidification. Scientific Reports.

[47] Liu H (2017). Columnar-to-equiaxed transition and euquiaxed grain alignment in directionally solidified Ni_3_Al alloy under an axial magnetic field. Metal. Mater. Trans. A.

[48] Schneider MC, Gu JP, Beckermann C, Boettinger WJ, Kattner UR (1997). Modeling of micro and macrosegregation and freckle formation in single-crystal nickel-base superalloy. Metal. Mater. Trans. A.

[49] Liang YJ, Li A, Cheng X, Pang XT, Wang HM (2016). Prediction of primary dendrite arm spacing during laser rapid directional solidification of single-crystal nickel-base superalloys. J. Alloys Compd..

[50] Guo, J.T. Materials science and engineering for superalloys vol. 1 (ed. Hou, S.S.) 89–103 (China science press, 2008).

[51] Reed, R.C. The superalloys fundamentals and applications (Trans. He, Y.H. *et al*., ed. Kong J. & Zhang C.L.) 147–150 (China machine press, 2016).

[52] Li HT (2016). Two-dimensional X-Ray diffraction to investigate γ/γ′ lattice misfits in different preparations of single-crystal nickel-based superalloy DD483. High Temp. Mater. Proc..

