# Supplementary material for: Improvement in creep life of a nickel-based single-crystal superalloy via composition homogeneity on the multiscales by magnetic-field-assisted directional solidification
Source: Sci Rep. 2018 Jan 23;8:1452. doi: 10.1038/s41598-018-19800-5 (PMC5780385; doi:10.1038/s41598-018-19800-5)
Supplement: Supplementary file 1 — Supplementary materials [file 41598_2018_19800_MOESM1_ESM.pdf]

Supplementary Materials

Improvement in creep life of a nickel-based single-crystal superalloy via composition homogeneity on the multiscales by magnetic-field-assisted directional solidification

Weili Ren [1, *], Chunlin Niu [1], Biao Ding [1], Yunbo Zhong [1*], Jianbo Yu [1], Zhongming Ren [1], Wenqing Liu [2], Liangpu Ren [3], Peter K. Liaw [4]

[1] State Key Laboratory of Advanced Special Steel, College of Materials Science and Engineering, Shanghai University, Shanghai 200072, PR China.

[2] Instrumental Analysis & Research Center, Shanghai University, Shanghai 200072, PR China.

[3] Microelctric Research and Development Center, Shanghai University, Shanghai 200072.

[4] Department of Materials Science and Engineering, The University of Tennessee, Knoxville, TN 37996, USA.

*Correspondence and requests for materials should be addressed to W.L.R. (email: wlren@staff.shu.edu.cn) or to Y.B.Z. (email: yunboz@ staff.shu.edu.cn).

1. Creep behavior at 250MPa/980℃.

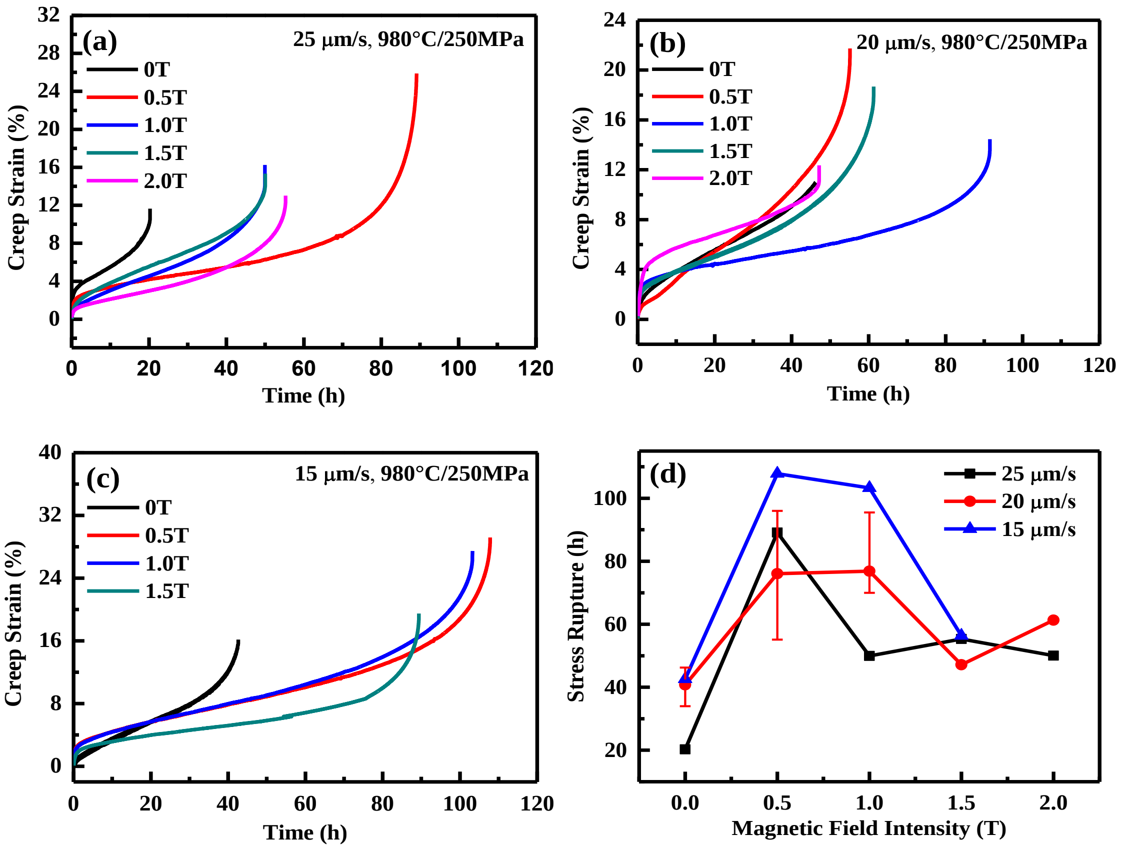

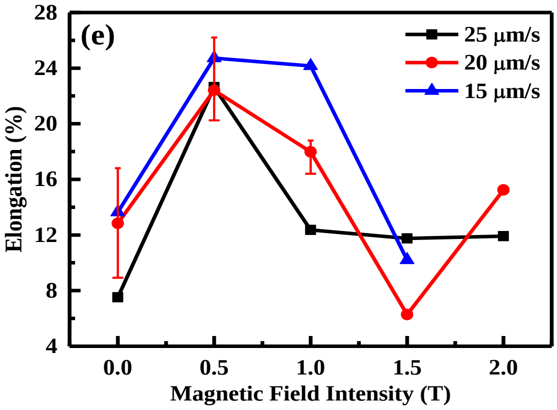

Figure 1. Creep behavior at 250MPa/980℃. (a ~ c) The creep strain as a function of time at the different withdraw velocities (15, 20, and 25 μm/s). (d) Creep-rupture life and (e) elongation with the magnetic-field intensity. The three specimens at the withdrawal velocity of 20 μm/s and the magnetic field of 0T, 0.5T, and 1T were conducted.

2. Solute distribution along longitudinal section of samples solidified at 15 μm/s.

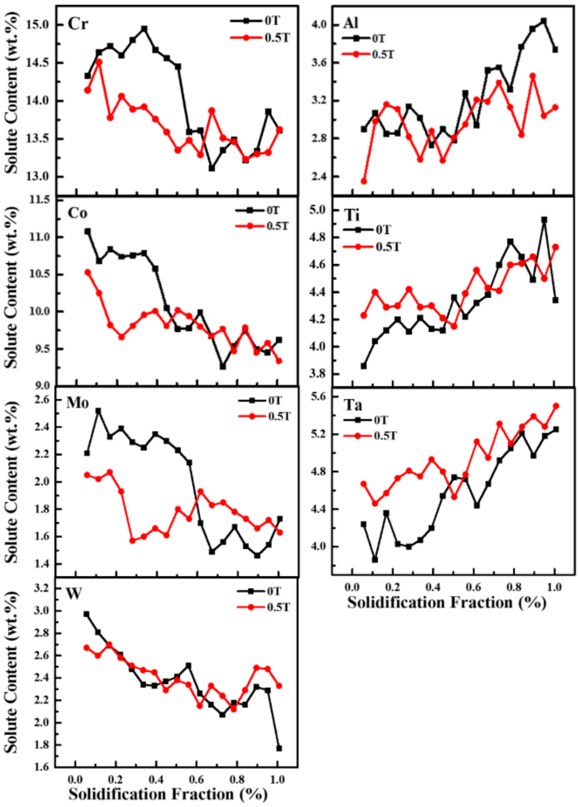

Figure 2. Solute distribution along the longitudinal section of the samples solidified at 15 μm/s. The alloying elements are marked at the top left corner in each subgraph.

3. Morphologies of carbide and γ/γ' eutectic in superalloys prepared at 15 μm/s.

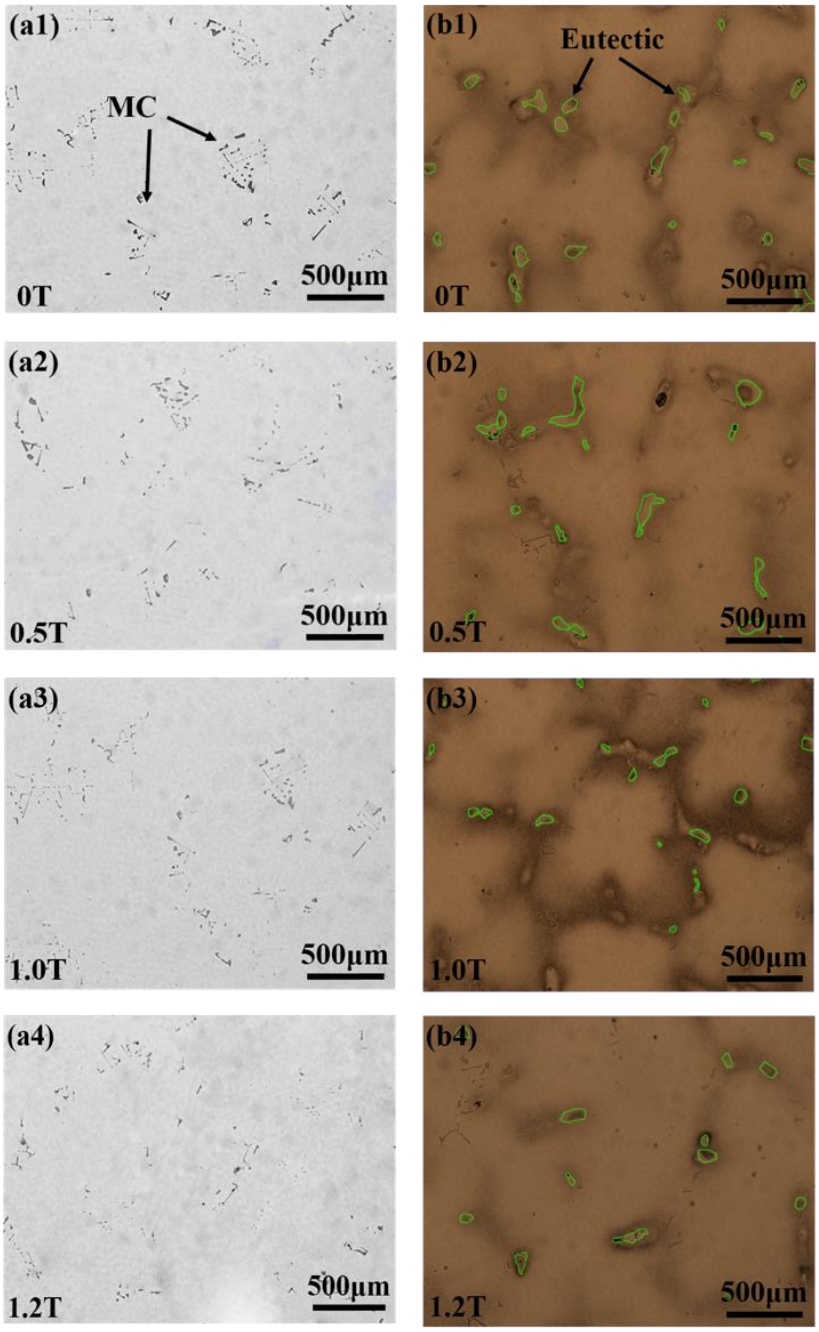

Figure 3. Precipitation phases in the superalloys at 15 μm/s. (a1 ~ a4) carbide phase. (b1 ~ b4) γ/γ' eutectic phase. The sample is from the 3 cm position below the completed solidification.

4. The initial morphology at the melted-unmelted interface.

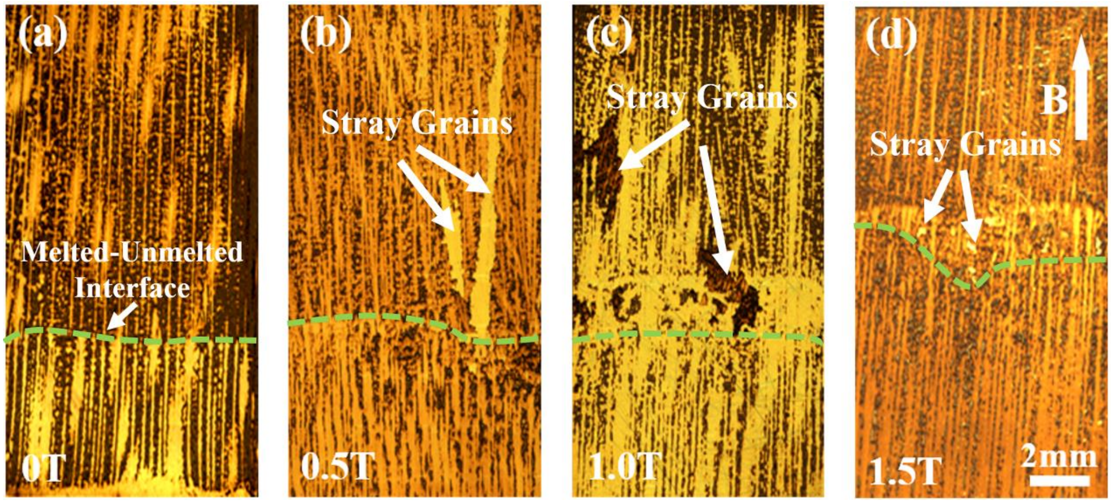

Figure 4. The initial morphology at the melted-unmelted interface. (a) 0T. (b) 0.5T. (c) 1.0T. (d) 1.5T. The stray grains formed at the melted-unmenlted interface (marked by the green-dashed line) of the superalloy during the initial directional solidification with the magnetic fields at the 25 μm/s.

5. The shape of liquid-solid interface.

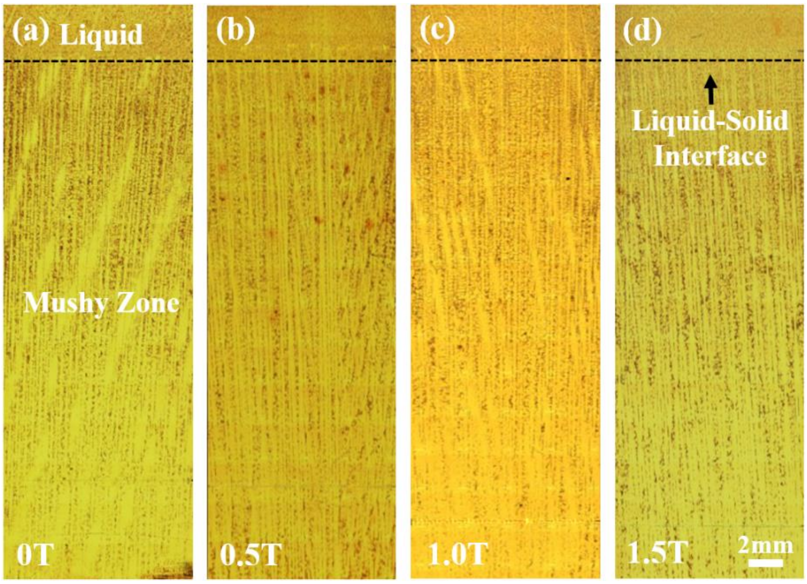

Figure 5. The liquid-solid interface shape of the superalloy at the quenched time when the samples were directionally solidified to 8 cm with the different magnetic fields at the 25 μm/s. (a) 0T. (b) 0.5T. (c) 1.0T. (d) 1.5T.

6. The crack and pore density.

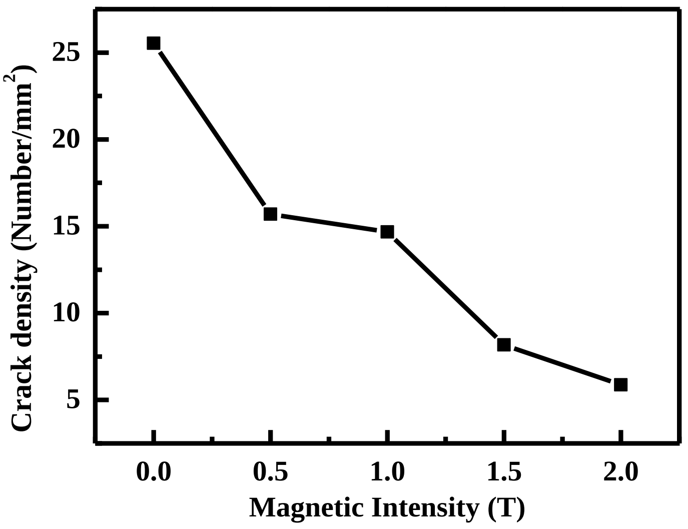

Figure 6. The crack and pore density in the area of 1.5 mm away the top of fracture surface on the longitudinal microstructure in the samples crept at 980°C/250MPa (solidified at 25 μm/s).

7. The temperature gradient in the melt.

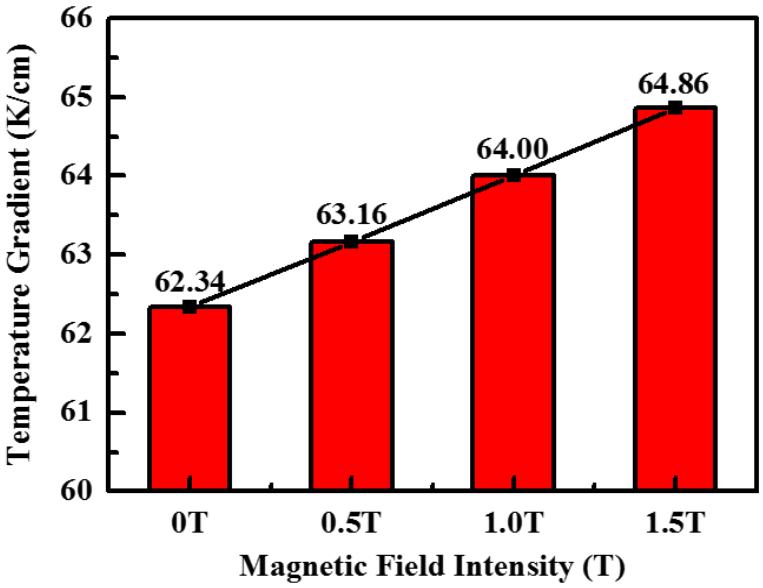

Figure 7. The temperature gradient in the melt with the different magnetic field intensities.

8. Numerical simulation.

The model Bridgman system is presented in Fig. 8. A 10-mm-diameter and 50-mm-tall melt cylinder were chosen. The initial temperature of the melt presents the gradient distribution of 20 K/cm. The alloy was Ni-40.38 wt.% Cu.

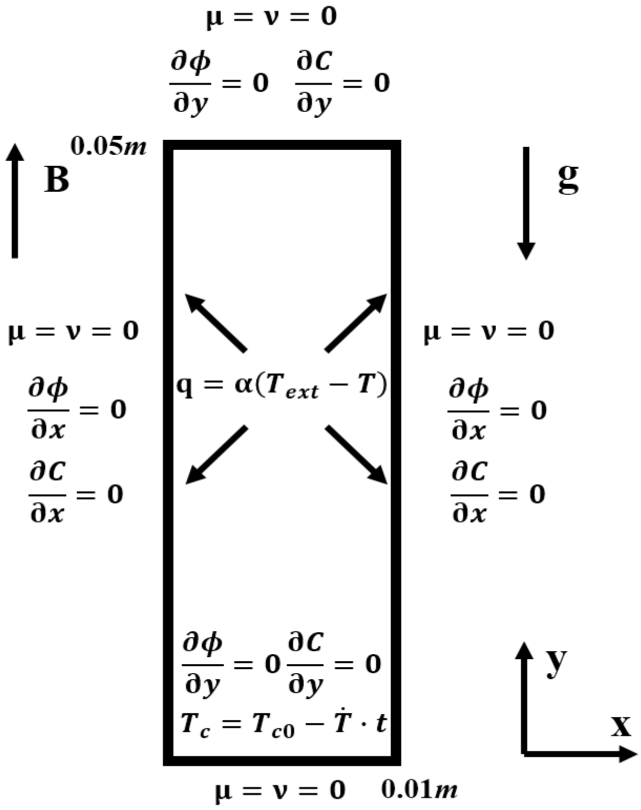

Figure 8. Schematic diagram and geometric definitions for the vertical Bridgman growth configuration. *B* is the magnetic field. *R* is the solidification rate.

The following assumptions were made:

1) Only the solid and liquid phases are present, i.e., no pores are formed.

2) The liquid is Newtonian and incompressible, and the flow is laminar.

3) The solid and liquid phases have the same thermal properties and densities.

4) There is no diffusion of solutes in the solid phase.

5) The thermal properties are constant, which allows the use of the Boussinesq approximation. Hence, the density is constant except in the body-force term of the momentum equation.

6) The solid is stationary. The liquid and solid concentrations at the interface are in the local equilibrium.

With these assumptions, the governing equations for the transport processes in the melt during crystal growth can be described by the conservation laws for the mass, momentum, energy, solute, and phase field as follows:

$$\nabla\cdot\left(\rho V\right)=0 \tag{1}$$

The *x*-axis component:

$$\frac{\partial}{\partial t}(\rho u)+\nabla\cdot(\rho V u)=\nabla\cdot(\mu_l\nabla u)-\frac{\mu_l}{K}u-\frac{\partial p}{\partial x}\text{-}\sigma_B B^2 u \tag{2}$$

The *y*-axis component:

$$\frac{\partial}{\partial t}(\rho v)+\nabla\cdot(\rho V v)=\nabla\cdot(\mu_l\nabla v)-\frac{\mu_l}{K}v-\frac{\partial p}{\partial y}+\rho g\left[\beta_T(T-T_{\mathrm{ref}})+\beta_c(c-c_{\mathrm{ref}})\right] \tag{3}$$

$$\frac{\partial T}{\partial t}+\nabla\cdot\left(VT\right)=\frac{\lambda}{\rho c_p}\nabla^2 T+\frac{L}{c_p}\frac{\partial\phi}{\partial t} \tag{4}$$

$$\frac{\partial C}{\partial t}+\nabla\cdot\left(VC\right)=\nabla\cdot\tilde{D}\left[\nabla C+\frac{1-k_p}{\phi+k_p(1-\phi)}C\nabla\phi\right] \tag{5}$$

$$\tilde{D}=D_s+(D_l-D_s)\frac{\phi}{\phi+k_p(1-\phi)} \tag{6}$$

$$\frac{\partial\phi}{\partial t}=M_\phi\varepsilon_\phi^2\left[\nabla^2\phi-\frac{2W_A}{\varepsilon_\phi^2}\phi\left(1-\phi\right)\left(1-2\phi\right)\right]-\frac{30M_\phi L(T_M-T+m_L C_l)}{T_M}\phi^2\left(1-\phi\right)^2 \tag{7}$$

In the above equations, ρ is the melt density, V is the velocity vector, μ is the x-axis

velocity, v is the y-axis velocity, t is the time, μ_l is the viscosity, K is the permeability, p is the pressure, σ_B is the electrical conductivity, B is the magnetic field intensity, β_T is the thermal-expansion coefficient, β_c is the solutal expansion coefficient, T_{ref} is the reference temperature, c_{ref} is the reference concentration, λ is the thermal conductivity, c_p is the specific heat, L is the latent heat, ϕ is the volume fraction of the solid, C is the average mixture concentration, k_p is the equilibrium partition coefficient, $\tilde{D}$ is a mixture diffusivity, D_l is the liquid's diffusivity, D_s is the solid's diffusivity, M_ϕ is the motilities related to the interface kinetic coefficient, ε_ϕ is the gradient entropy coefficients related to the phase field, ϕ, m_L is the liquid's slope, C_l is the liquid's concentration, T_M is the pure component melting point, and W_A is an energy hump, which is a constant.

The temperature-concentration relation is:

$$T = T_M + m_L \, (\tag{8}$$

where m_L is the liquid's slope, and C_l is the liquid's concentration.

The boundary and initial conditions of the calculated domain are described in Fig. 8. The given value of the parameters of Ni-40.38 wt.%.Cu is in Table 1.

Figure 9 shows the flow field in the melt solidified during the different magnetic fields in the Ni-40.38 wt%.Cu alloy. The flow shows the structure of the axial symmetry in all the samples. However, the MHD effect changes the flow-cell number on each side, as indicated by the white dash-line rectangle. There is each flow cell in the lower and upper regions of the melt without the magnetic field in Fig. 9(a). The MHD effect merges the two cell into one. Figure 9(b) is in the condition of being merged. Figure 9(c) shows the complete-mergement condition. With increasing the magnetic-field intensity, the cell

width becomes narrower as in Fig. 9(e). The convection velocity is also decreased with the MHD effect in Fig. 10. For example, the velocity in the melt is decreased by 30% at the middle time with the 2T magnetic field.

Table 1. Parameters of the Ni-40.38 wt.%Cu alloy used in the simulation.

Parameter	Symbol (Unit)	Value
Density	ρ (Kg/m^3)	8,850
Melting point	T_M (K)	1,595
Initial concentration.	C_0 (%)	0.40
Viscosity	μ_l $(Pa{\cdot}s)$	1×10^{-3}
Diffusion coefficient in liquid	D_l (cm^2/s)	1×10^{-5}
Diffusion coefficient in solid	D_s (cm^2/s)	1×10^{-9}
The slope of liquid line	m_L (ºC/%)	357
Solidus temperature	T_S (K)	1,559
Thermal-expansion coefficient	β_T (K^{-1})	-1×10^{-4}
Solutal-expansion coefficient	β_c (1/%)	-1×10^{-3}
Thermal conductivity	λ $(J/m{\cdot}s{\cdot}k)$	100
Specific heat	c_p $(J/kg{\cdot}k)$	483
Latent heat	L (J/m^3)	2.1×10^9
Equilibrium-partition coefficient	k_p	0.75

The effect of MHD on the temperature field in the Ni-40.38 wt.% Cu alloy is not so obvious (Fig. 11). However, it make the temperature gradient in the liquid increase (Fig. 12). As we know, MHD could suppress the melt flow, which would increase the temperature gradient. On the contrary the thermoelectromagnetic convection (TEMC) induces a flow in the melt and further decreases the temperature. Therefore, the MHD control the convection on the macro scale.

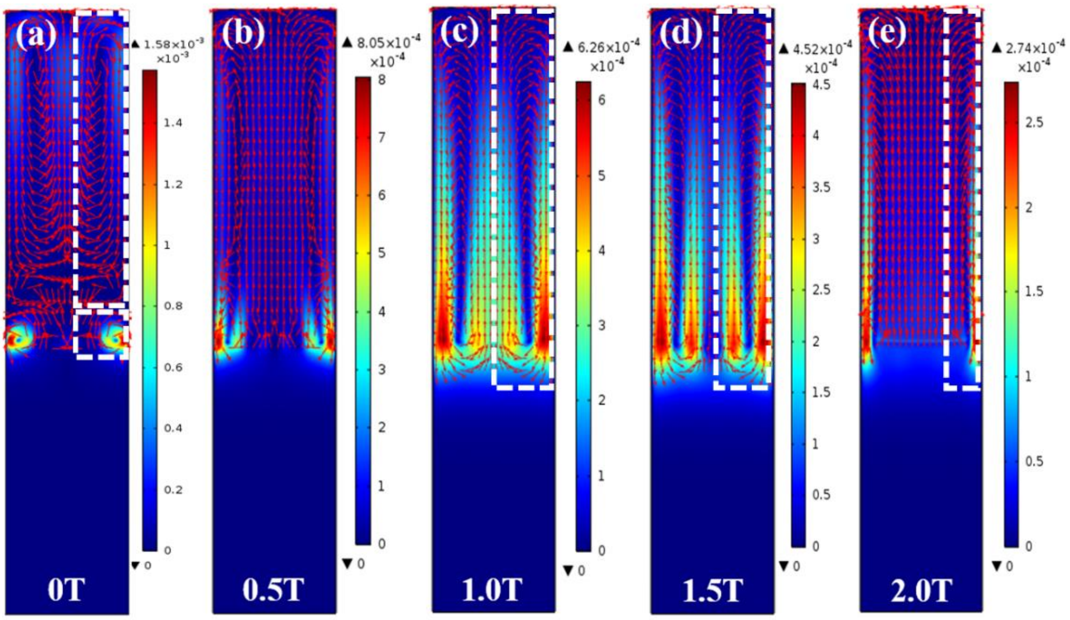

Figure 9. Flow field in Ni-40.38 wt%.Cu alloy solidified in different magnetic fields. (a) 0T. (b) 0.5T. (c) 1T. (d) 1.5T. (e) 2T. The MHD effect merges the two cells [as indicated by the white dash-line rectangle in (a)] into one [as in (c)] and make the cell width narrow [as in (e)].

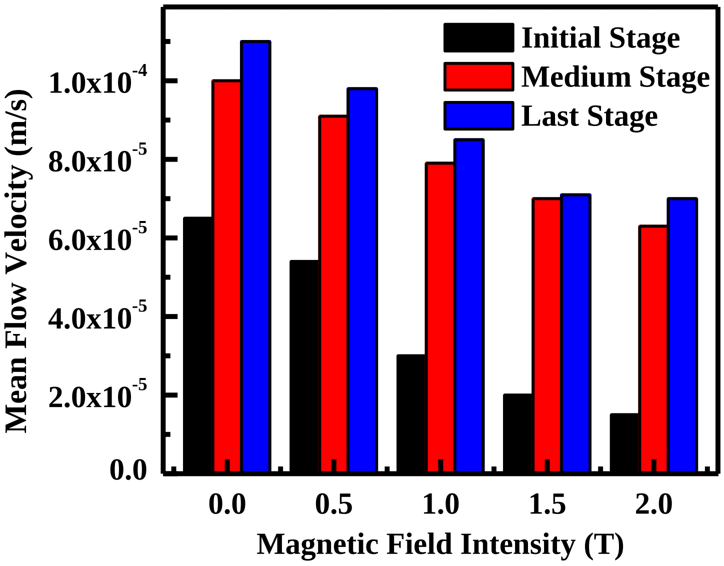

Figure 10. Mean convection velocity in the melt during the different solidification periods and with the various magnetic field intensities.

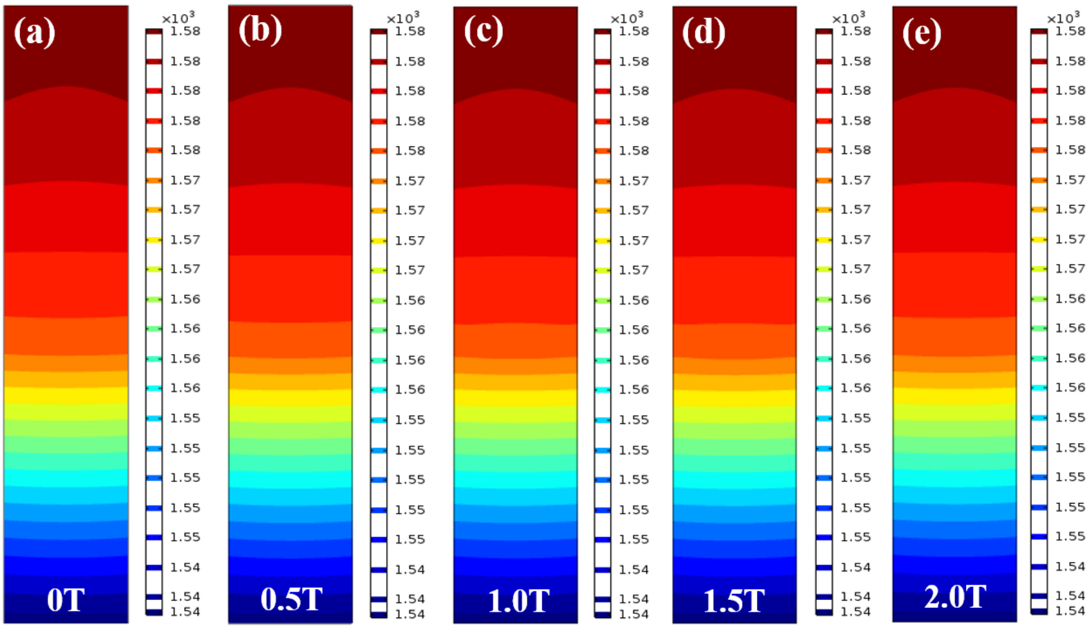

Figure 11. Temperature distribution during solidification in the Ni-40.38 wt.%Cu alloy with different magnetic fields. (a) 0T. (b) 0.5T. (c) 1T. (d) 1.5T. (e) 2T.

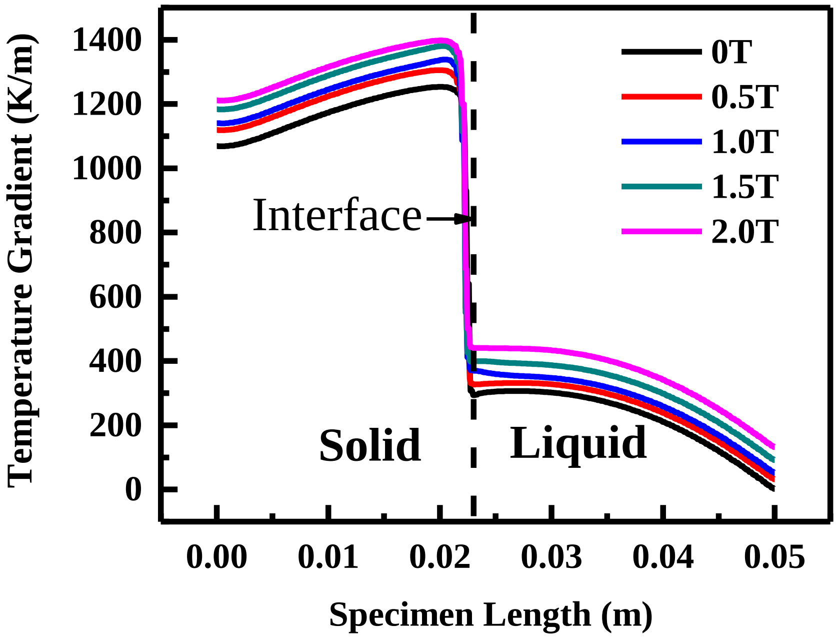

Figure 12. Temperature gradient along the center longitudinal line in the samples during the middle solidification period with the different magnetic field intensities.

The effect of MHD on the convection and temperature fields further modifies the solute distribution in the whole macro-sample. The change of the convection structure, the decrease in the convection velocity, and the increase in the temperature gradient in the liquid make the solute present a more homogeneous distribution in the alloy.
